# Supplementary figures and images for: A genome-wide association study in Indian wild rice accessions for resistance to the root-knot nematode Meloidogyne graminicola
Source: PLoS One. 2020 Sep 22;15(9):e0239085. doi: 10.1371/journal.pone.0239085 (PMC7508375; doi:10.1371/journal.pone.0239085)

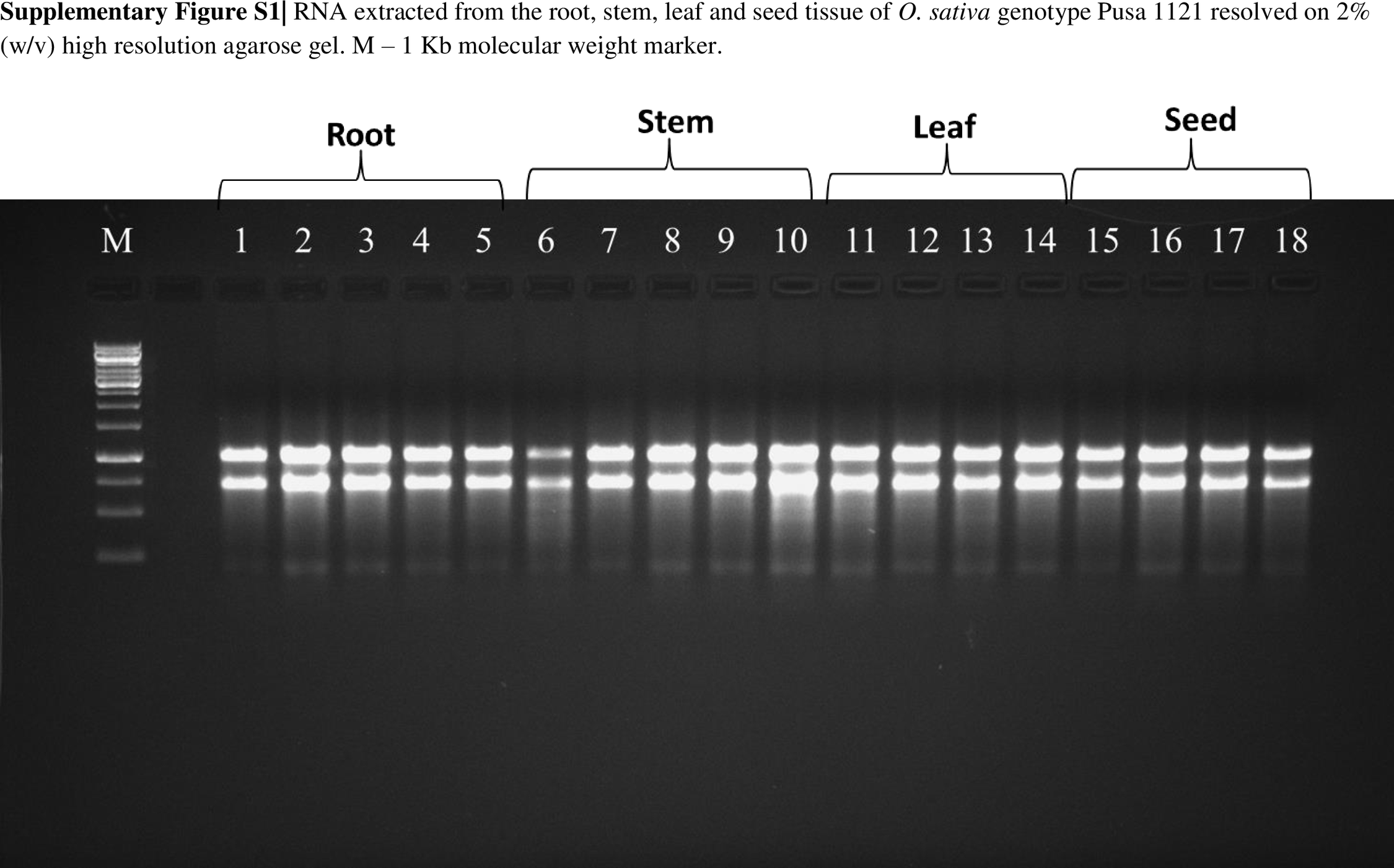

Supplement: S1 Fig — M– 1 Kb molecular weight marker. (TIF) [file pone.0239085.s001.tif]

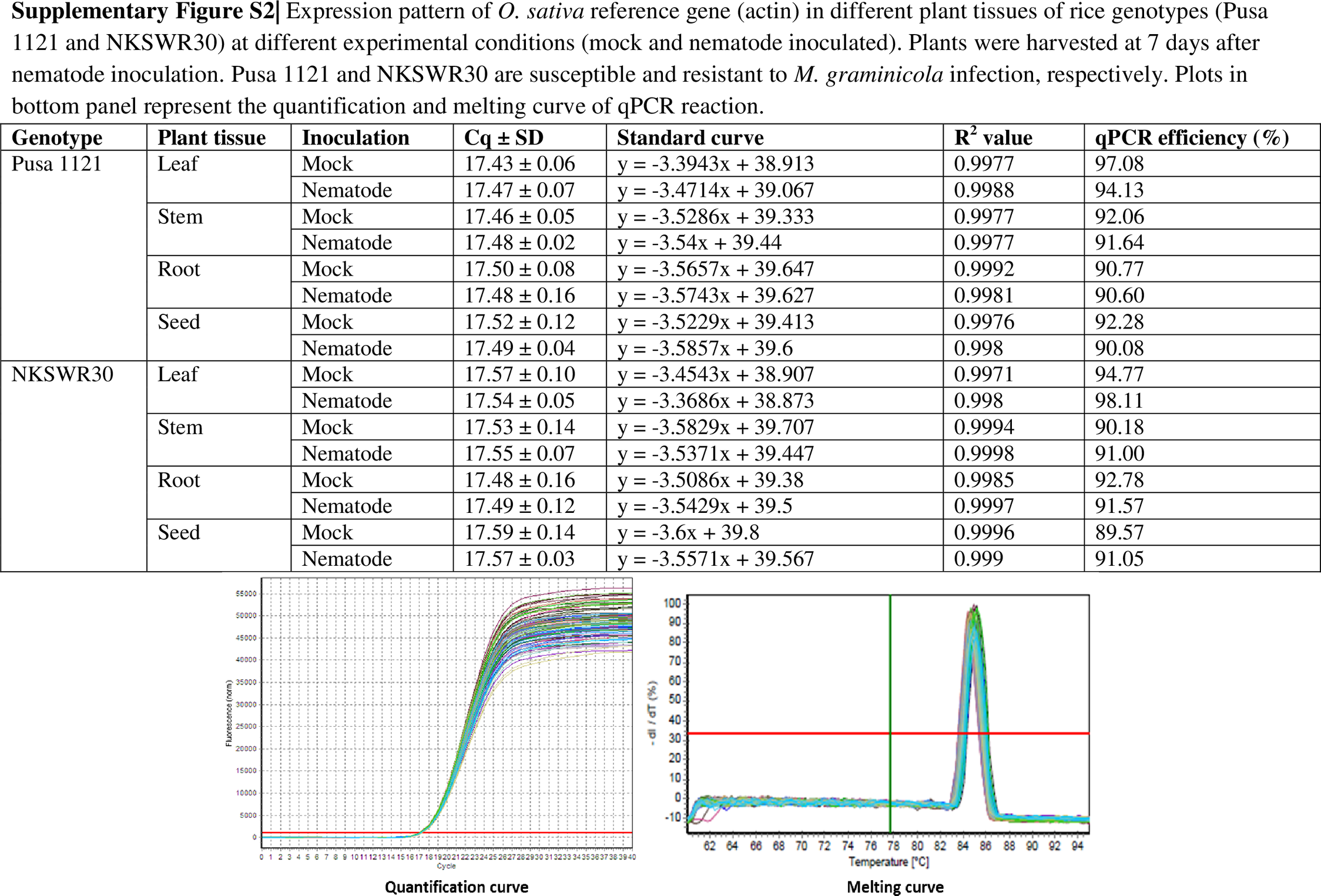

Supplement: S2 Fig — Plants were harvested at 7 days after nematode inoculation. Pusa 1121 and NKSWR30 are susceptible and resistant to M. graminicola infection, respectively. Plots in bottom panel represent the quantification and melting curve of qPCR reaction. (TIF) [file pone.0239085.s002.tif]

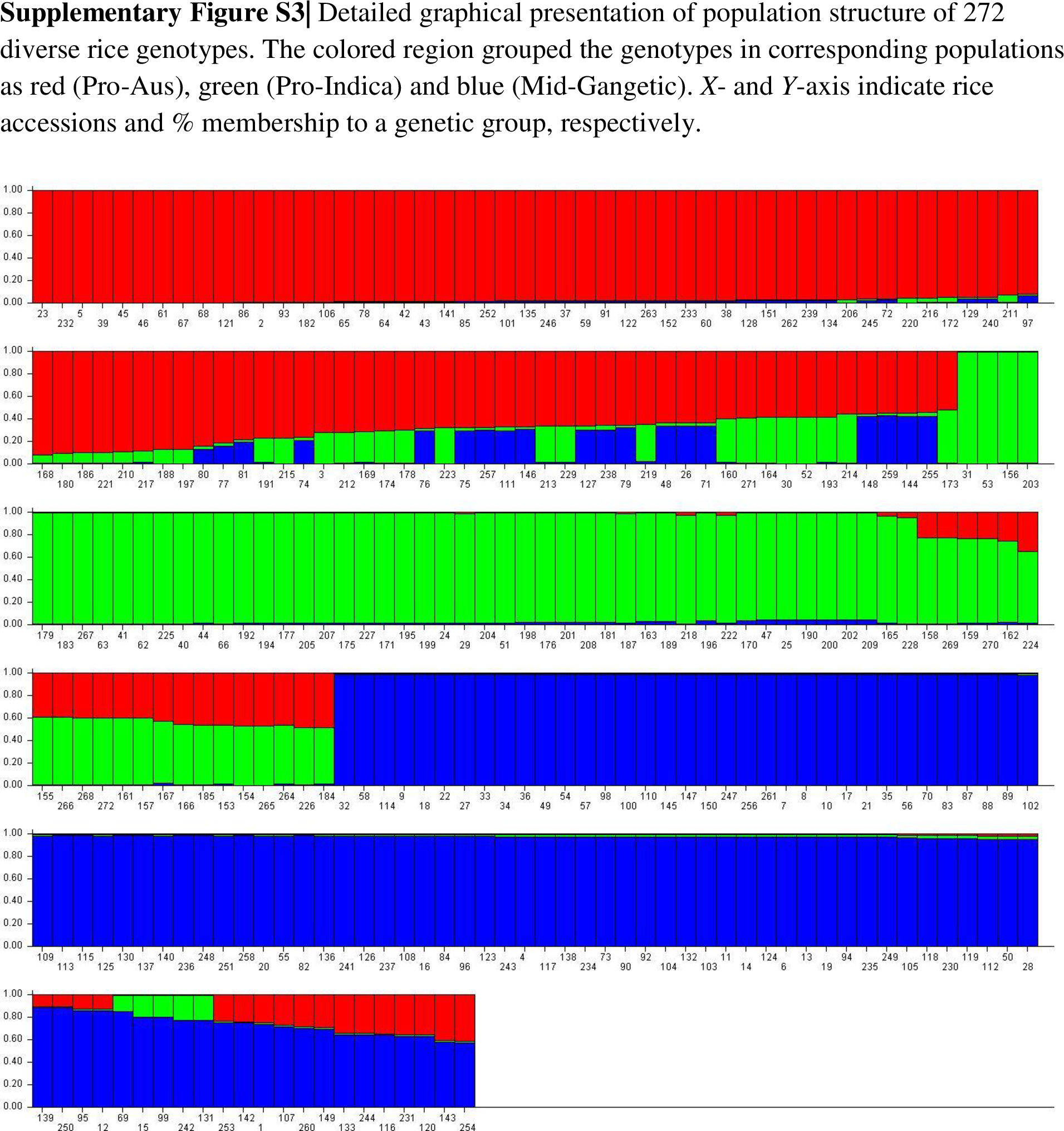

Supplement: S3 Fig — The colored region grouped the genotypes in corresponding populations as red (Pro-Aus), green (Pro-Indica) and blue (Mid-Gangetic). X- and Y-axis indicate rice accessions and % membership to a genetic group, respectively. (TIF) [file pone.0239085.s003.tif]

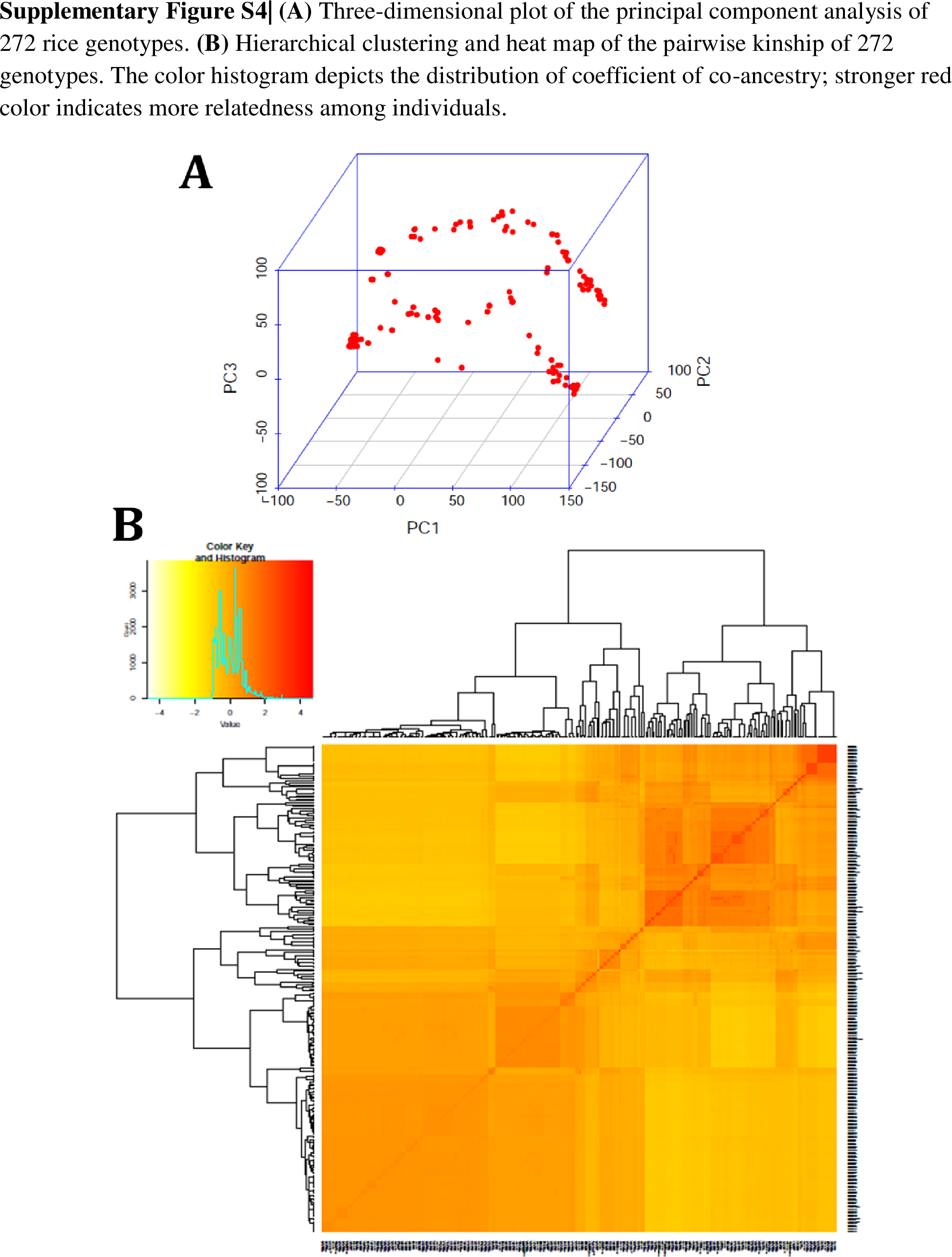

Supplement: S4 Fig — (A) Three-dimensional plot of the principal component analysis of 272 rice genotypes. (B) Hierarchical clustering and heat map of the pairwise kinship of 272 genotypes. The color histogram depicts the distribution of coefficient of co-ancestry; stronger red color indicates more relatedness among individuals. (TIF) [file pone.0239085.s004.tif]
