# Supplementary material for: A genome-wide association study in Indian wild rice accessions for resistance to the root-knot nematode Meloidogyne graminicola
Source: PLoS One. 2020 Sep 22;15(9):e0239085. doi: 10.1371/journal.pone.0239085 (PMC7508375; doi:10.1371/journal.pone.0239085)
Supplement: S1 Table — (PDF) [file pone.0239085.s006.pdf]

Supplementary Table S1| List of 272 rice genotypes used in the present study including wild rice or landraces (prefixed as NKSWR/IC) or improved cultivars collected from the different agro-climatic zones of India.

S. No.	Accessions	Species	Agro-climatic zone	States	District	Sub-population
1	NKSWR10	*Oryza nivara*	Mid-Gangetic Plains (MGP)	Uttar Pradesh	Mirzapur	Pro-Aus
2	NKSWR101	*Oryza spontanea*	Mid-Gangetic Plains (MGP)	Uttar Pradesh	Ballia	Mid-Gangatic
3	NKSWR102	*Oryza nivara*	Mid-Gangetic Plains (MGP)	Uttar Pradesh	Ghazipur	Mid-Gangatic
4	NKSWR104	*Oryza nivara*	Mid-Gangetic Plains (MGP)	Bihar	Kaimur	Mid-Gangatic
5	NKSWR105	*Oryza nivara*	Mid-Gangetic Plains (MGP)	Bihar	Bhabhua	Mid-Gangatic
6	NKSWR108	*Oryza nivara*	Mid-Gangetic Plains (MGP)	Bihar	Kaimur	Mid-Gangatic
7	NKSWR109	*Oryza nivara*	Mid-Gangetic Plains (MGP)	Bihar	Kaimur	Mid-Gangatic
8	NKSWR11	*Oryza nivara*	Mid-Gangetic Plains (MGP)	Uttar Pradesh	Mirzapur	Mid-Gangatic
9	NKSWR110	*Oryza nivara*	Mid-Gangetic Plains (MGP)	Bihar	Kaimur	Mid-Gangatic
10	NKSWR112	*Oryza nivara*	Mid-Gangetic Plains (MGP)	Bihar	Kaimur	Mid-Gangatic
11	NKSWR113	*Oryza nivara*	Mid-Gangetic Plains (MGP)	Bihar	Kaimur	Mid-Gangatic
12	NKSWR114	*Oryza nivara*	Mid-Gangetic Plains (MGP)	Bihar	Rohtas	Mid-Gangatic
13	NKSWR115	*Oryza nivara*	Mid-Gangetic Plains (MGP)	Bihar	Rohtas	Mid-Gangatic
14	NKSWR116	*Oryza rufipogon*	Mid-Gangetic Plains (MGP)	Bihar	Rohtas	Mid-Gangatic
15	NKSWR117	*Oryza nivara*	Mid-Gangetic Plains (MGP)	Bihar	Rohtas	Mid-Gangatic
16	NKSWR118	*Oryza nivara*	Mid-Gangetic Plains (MGP)	Bihar	Rohtas	Mid-Gangatic
17	NKSWR119	*Oryza nivara*	Mid-Gangetic Plains (MGP)	Bihar	Buxar	Mid-Gangatic
18	NKSWR12	*Oryza spontanea*	Mid-Gangetic Plains (MGP)	Uttar Pradesh	Mirzapur	Mid-Gangatic
19	NKSWR120	*Oryza spontanea*	Mid-Gangetic Plains (MGP)	Bihar	Buxar	Mid-Gangatic
20	NKSWR121	*Oryza nivara*	Mid-Gangetic Plains (MGP)	Bihar	Buxar	Mid-Gangatic
21	NKSWR122	*Oryza nivara*	Mid-Gangetic Plains (MGP)	Bihar	Bhojpur	Mid-Gangatic
22	NKSWR123	*Oryza nivara*	Mid-Gangetic Plains (MGP)	Bihar	Bhojpur	Mid-Gangatic
23	NKSWR124	*Oryza nivara*	Mid-Gangetic Plains (MGP)	Bihar	Bhojpur	Mid-Gangatic
24	NKSWR125	*Oryza nivara*	Mid-Gangetic Plains (MGP)	Bihar	Bhojpur	Mid-Gangatic
25	NKSWR126	*Oryza nivara*	Mid-Gangetic Plains (MGP)	Bihar	Bhojpur	Mid-Gangatic

26	NKSWR127	*Oryza nivara*	Mid-Gangetic Plains (MGP)	Bihar	Rohtas	Mid-Gangatic
27	NKSWR128	*Oryza nivara*	Mid-Gangetic Plains (MGP)	Bihar	Rohtas	Mid-Gangatic
28	NKSWR13	*Oryza nivara*	Mid-Gangetic Plains (MGP)	Uttar Pradesh	Mirzapur	Pro-Aus
29	NKSWR132	*Oryza nivara*	Mid-Gangetic Plains (MGP)	Bihar	Rohtas	Mid-Gangatic
30	NKSWR133	*Oryza nivara*	Mid-Gangetic Plains (MGP)	Bihar	Aurangabad	Mid-Gangatic
31	NKSWR134	*Oryza nivara*	Mid-Gangetic Plains (MGP)	Bihar	Aurangabad	Mid-Gangatic
32	NKSWR136	*Oryza nivara*	Mid-Gangetic Plains (MGP)	Bihar	Aurangabad	Pro-Aus
33	NKSWR139	*Oryza rufipogon*	Mid-Gangetic Plains (MGP)	Bihar	Aurangabad	Mid-Gangatic
34	NKSWR14	*Oryza nivara*	Mid-Gangetic Plains (MGP)	Uttar Pradesh	Mirzapur	Mid-Gangatic
35	NKSWR140	*Oryza nivara*	Mid-Gangetic Plains (MGP)	Bihar	Aurangabad	Mid-Gangatic
36	NKSWR141	*Oryza nivara*	Mid-Gangetic Plains (MGP)	Bihar	Aurangabad	Mid-Gangatic
37	NKSWR142	*Oryza nivara*	Mid-Gangetic Plains (MGP)	Bihar	Gaya	Mid-Gangatic
38	NKSWR144	*Oryza nivara*	Mid-Gangetic Plains (MGP)	Bihar	Gaya	Mid-Gangatic
39	NKSWR145	*Oryza nivara*	Mid-Gangetic Plains (MGP)	Bihar	Gaya	Mid-Gangatic
40	NKSWR146	*Oryza rufipogon*	Mid-Gangetic Plains (MGP)	Bihar	Gaya	Mid-Gangatic
41	NKSWR148	*Oryza nivara*	Mid-Gangetic Plains (MGP)	Bihar	Jehanabad	Mid-Gangatic
42	NKSWR149	*Oryza nivara*	Mid-Gangetic Plains (MGP)	Bihar	Patna	Mid-Gangatic
43	NKSWR15	*Oryza nivara*	Mid-Gangetic Plains (MGP)	Uttar Pradesh	Mirzapur	Pro-Aus
44	NKSWR150	*Oryza nivara*	Mid-Gangetic Plains (MGP)	Bihar	Patna	Mid-Gangatic
45	NKSWR151	*Oryza rufipogon*	Mid-Gangetic Plains (MGP)	Bihar	Patna	Pro-Indica
46	NKSWR152	*Oryza nivara*	Mid-Gangetic Plains (MGP)	Bihar	Patna	Pro-Indica
47	NKSWR153	*Oryza nivara*	Mid-Gangetic Plains (MGP)	Bihar	Patna	Mid-Gangatic
48	NKSWR154	*Oryza nivara*	Mid-Gangetic Plains (MGP)	Bihar	Patna	Mid-Gangatic
49	NKSWR155	*Oryza nivara*	Mid-Gangetic Plains (MGP)	Bihar	Bhojpur	Mid-Gangatic
50	NKSWR156	*Oryza nivara*	Mid-Gangetic Plains (MGP)	Bihar	Bhojpur	Mid-Gangatic
51	NKSWR157	*Oryza nivara*	Mid-Gangetic Plains (MGP)	Bihar	Bhojpur	Mid-Gangatic
52	NKSWR159	*Oryza nivara*	Mid-Gangetic Plains (MGP)	Bihar	Buxar	Mid-Gangatic
53	NKSWR16	*Oryza nivara*	Mid-Gangetic Plains (MGP)	Uttar Pradesh	Mirzapur	Mid-Gangatic
54	NKSWR160	*Oryza nivara*	Mid-Gangetic Plains (MGP)	Bihar	Buxar	Mid-Gangatic

55	NKSWR161	*Oryza nivara*	Upper Gangetic Plains (UGP)	Uttar Pradesh	Gautam budha nagar	Pro-Aus
56	NKSWR162	*Oryza nivara*	Upper Gangetic Plains (UGP)	Uttar Pradesh	Gautam budha nagar	Pro-Aus
57	NKSWR163	*Oryza nivara*	Upper Gangetic Plains (UGP)	Uttar Pradesh	Gautam budha nagar	Pro-Aus
58	NKSWR164	*Oryza spontanea*	Upper Gangetic Plains (UGP)	Uttar Pradesh	Aligarh	Pro-Aus
59	NKSWR169	*Oryza nivara*	Western Himalayan Regions (WHR)	Himachal Pradesh	Mandi	Pro-Aus
60	NKSWR17	*Oryza spontanea*	Mid-Gangetic Plains (MGP)	Uttar Pradesh	Sonbhadra	Pro-Aus
61	NKSWR173	*Oryza nivara*	Western Himalayan Regions (WHR)	Himachal Pradesh	Kangra	Pro-Indica
62	NKSWR174	*Oryza spontanea*	Western Himalayan Regions (WHR)	Himachal Pradesh	Kangra	Pro-Aus
63	NKSWR179	*Oryza nivara*	Western Himalayan Regions (WHR)	Himachal Pradesh	Kangra	Pro-Aus
64	NKSWR18	*Oryza nivara*	Mid-Gangetic Plains (MGP)	Uttar Pradesh	Sonbhadra	Pro-Aus
65	NKSWR183	*Oryza nivara*	Western Himalayan Regions (WHR)	Himachal Pradesh	Chamba	Pro-Aus
66	NKSWR184	*Oryza spontanea*	Western Himalayan Regions (WHR)	Himachal Pradesh	Chamba	Pro-Aus
67	NKSWR186	*Oryza nivara*	Western Himalayan Regions (WHR)	Himachal Pradesh	Kangra	Pro-Aus
68	NKSWR187	*Oryza nivara*	Upper Gangetic Plains (UGP)	Uttar Pradesh	Sitapur	Pro-Aus
69	NKSWR188	*Oryza nivara*	Upper Gangetic Plains (UGP)	Uttar Pradesh	Sitapur	Pro-Aus
70	NKSWR189	*Oryza nivara*	Upper Gangetic Plains (UGP)	Uttar Pradesh	Sitapur	Pro-Aus
71	NKSWR19	*Oryza nivara*	Mid-Gangetic Plains (MGP)	Uttar Pradesh	Sonbhadra	Mid-Gangatic
72	NKSWR190	*Oryza nivara*	Upper Gangetic Plains (UGP)	Uttar Pradesh	Sitapur	Pro-Aus
73	NKSWR191	*Oryza nivara*	Upper Gangetic Plains (UGP)	Uttar Pradesh	Hardoi	Pro-Aus
74	NKSWR192	*Oryza nivara*	Upper Gangetic Plains (UGP)	Uttar Pradesh	Unnao	Pro-Aus
75	NKSWR196	*Oryza nivara*	Upper Gangetic Plains (UGP)	Uttar Pradesh	Unnao	Pro-Aus
76	NKSWR197	*Oryza rufipogon*	Upper Gangetic Plains (UGP)	Uttar Pradesh	Unnao	Pro-Aus
77	NKSWR199	*Oryza nivara*	Upper Gangetic Plains (UGP)	Uttar Pradesh	Unnao	Pro-Indica
78	NKSWR200	*Oryza nivara*	Upper Gangetic Plains (UGP)	Uttar Pradesh	Lucknow	Pro-Aus
79	NKSWR202	*Oryza nivara*	Mid-Gangetic Plains (MGP)	Uttar Pradesh		Pro-Aus

80	NKSWR205	*Oryza nivara*	Mid-Gangetic Plains (MGP)	Uttar Pradesh	Basti	Pro-Aus
81	NKSWR207	*Oryza nivara*	Mid-Gangetic Plains (MGP)	Uttar Pradesh	Gorakhpur	Pro-Aus
82	NKSWR21	*Oryza spontanea*	Mid-Gangetic Plains (MGP)	Uttar Pradesh	Sonbhadra	Mid-Gangatic
83	NKSWR210	*Oryza nivara*	Mid-Gangetic Plains (MGP)	Uttar Pradesh	Kushi Nagar	Pro-Aus
84	NKSWR218	*Oryza nivara*	Mid-Gangetic Plains (MGP)	Bihar	East Champaran	Pro-Aus
85	NKSWR22	*Oryza nivara*	Mid-Gangetic Plains (MGP)	Uttar Pradesh	Sonbhadra	Pro-Aus
86	NKSWR220	*Oryza nivara*	Mid-Gangetic Plains (MGP)	Bihar	Sitamarhi	Pro-Aus
87	NKSWR221	*Oryza nivara*	Mid-Gangetic Plains (MGP)	Bihar	Madhubani	Pro-Aus
88	NKSWR222	*Oryza nivara*	Mid-Gangetic Plains (MGP)	Bihar	Madhubani	Pro-Aus
89	NKSWR223	*Oryza nivara*	Mid-Gangetic Plains (MGP)	Bihar	Vaishali	Pro-Aus
90	NKSWR224	*Oryza nivara*	Mid-Gangetic Plains (MGP)	Bihar	Patna	Pro-Aus
91	NKSWR226	*Oryza nivara*	Mid-Gangetic Plains (MGP)	Bihar	Patna	Pro-Aus
92	NKSWR227	*Oryza nivara*	Western Coastal Plains and ghats (WCP)	Gujarat	Navsari	Pro-Aus
93	NKSWR228	*Oryza nivara*	Western Coastal Plains and ghats (WCP)	Gujarat	Navsari	Pro-Aus
94	NKSWR229	*Oryza nivara*	Western Coastal Plains and ghats (WCP)	Gujarat	Navsari	Pro-Aus
95	NKSWR23	*Oryza nivara*	Mid-Gangetic Plains (MGP)	Uttar Pradesh	Sonbhadra	Pro-Aus
96	NKSWR230	*Oryza nivara*	Western Coastal Plains and ghats (WCP)	Gujarat	Navsari	Pro-Aus
97	NKSWR232	*Oryza spontanea*	Western Coastal Plains and ghats (WCP)	Gujarat	Navsari	Pro-Aus
98	NKSWR233	*Oryza nivara*	Western Coastal Plains and ghats (WCP)	Gujarat	Navsari	Pro-Aus
99	NKSWR234	*Oryza rufipogon*	Western Coastal Plains and ghats (WCP)	Gujarat	Navsari	Pro-Indica
100	NKSWR235	*Oryza nivara*	Western Coastal Plains and ghats (WCP)	Gujarat	Navsari	Pro-Aus
101	NKSWR236	*Oryza nivara*	Western Coastal Plains and ghats (WCP)	Gujarat	Surat	Pro-Aus
102	NKSWR239	*Oryza rufipogon*	Western Coastal Plains and ghats (WCP)	Gujarat	Navsari	Pro-Aus
103	NKSWR24	*Oryza nivara*	Mid-Gangetic Plains (MGP)	Uttar Pradesh	Sonbhadra	Pro-Aus
104	NKSWR241	*Oryza nivara*	Western Coastal Plains and ghats (WCP)	Gujarat	Navsari	Pro-Aus
105	NKSWR242	*Oryza nivara*	Western Coastal Plains and ghats (WCP)	Gujarat	Navsari	Pro-Aus
106	NKSWR243	*Oryza nivara*	Western Himalayan Regions (WHR)	Uttarakhand	Dehradun	Pro-Aus
107	NKSWR245	*Oryza nivara*	Western Himalayan Regions (WHR)	Uttarakhand	Dehradun	Pro-Aus
108	NKSWR246	*Oryza spontanea*	Western Himalayan Regions (WHR)	Uttarakhand	Dehradun	Pro-Aus

109	NKSWR247	*Oryza spontanea*	Western Himalayan Regions (WHR)	Uttarakhand	Dehradun	Pro-Aus
110	NKSWR248	*Oryza spontanea*	Western Himalayan Regions (WHR)	Uttarakhand	Dehradun	Mid-Gangatic
111	NKSWR249	*Oryza nivara*	Western Himalayan Regions (WHR)	Uttarakhand	Dehradun	Mid-Gangatic
112	NKSWR25	*Oryza nivara*	Mid-Gangetic Plains (MGP)	Uttar Pradesh	Chandauli	Mid-Gangatic
113	NKSWR250	*Oryza spontanea*	Western Himalayan Regions (WHR)	Uttarakhand	Dehradun	Mid-Gangatic
114	NKSWR251	*Oryza nivara*	Gujrat Plain and Hills (GPH)	Gujarat	Kheda	Mid-Gangatic
115	NKSWR252	*Oryza nivara*	Gujrat Plain and Hills (GPH)	Gujarat	Kheda	Mid-Gangatic
116	NKSWR253	*Oryza spontanea*	Gujrat Plain and Hills (GPH)	Gujarat	Kheda	Mid-Gangatic
117	NKSWR254	*Oryza nivara*	Gujrat Plain and Hills (GPH)	Gujarat	Kheda	Pro-Aus
118	NKSWR255	*Oryza nivara*	Gujrat Plain and Hills (GPH)	Gujarat	Kheda	Mid-Gangatic
119	NKSWR257	*Oryza nivara*	Gujrat Plain and Hills (GPH)	Gujarat	Ahmedabad	Pro-Aus
120	NKSWR258	*Oryza nivara*	Gujrat Plain and Hills (GPH)	Gujarat	Ahmedabad	Pro-Aus
121	NKSWR259	*Oryza nivara*	Gujrat Plain and Hills (GPH)	Gujarat	Aanad	Pro-Aus
122	NKSWR260	*Oryza nivara*	Gujrat Plain and Hills (GPH)	Gujarat	Vadodara	Pro-Aus
123	NKSWR261	*Oryza spontanea*	Gujrat Plain and Hills (GPH)	Gujarat	Vadodara	Pro-Aus
124	NKSWR262	*Oryza nivara*	Gujrat Plain and Hills (GPH)	Gujarat	Vadodara	Pro-Aus
125	NKSWR263	*Oryza spontanea*	Gujrat Plain and Hills (GPH)	Gujarat	Vadodara	Pro-Aus
126	NKSWR264	*Oryza nivara*	Gujrat Plain and Hills (GPH)	Gujarat	Vadodara	Pro-Aus
127	NKSWR265	*Oryza nivara*	Gujrat Plain and Hills (GPH)	Gujarat	Vadodara	Pro-Aus
128	NKSWR269	*Oryza spontanea*	Gujrat Plain and Hills (GPH)	Gujarat	Vadodara	Pro-Aus
129	NKSWR27	*Oryza spontanea*	Mid-Gangetic Plains (MGP)	Uttar Pradesh	Chandauli	Pro-Aus
130	NKSWR28	*Oryza spontanea*	Mid-Gangetic Plains (MGP)	Uttar Pradesh	Chandauli	Mid-Gangatic
131	NKSWR29	*Oryza nivara*	Mid-Gangetic Plains (MGP)	Uttar Pradesh	Chandauli	Mid-Gangatic
132	NKSWR3	*Oryza nivara*	Mid-Gangetic Plains (MGP)	Uttar Pradesh	Mirzapur	Pro-Aus
133	NKSWR30	*Oryza nivara*	Mid-Gangetic Plains (MGP)	Uttar Pradesh	Chandauli	Pro-Aus
134	NKSWR302	*Oryza nivara*	Western Coastal Plains and ghats (WCP)	Goa	North Goa	Pro-Aus
135	NKSWR304	*Oryza nivara*	Western Coastal Plains and ghats (WCP)	Goa	North Goa	Pro-Indica
136	NKSWR306	*Oryza spontanea*	Western Coastal Plains and ghats (WCP)	Goa	North Goa	Pro-Aus
137	NKSWR307	*Oryza nivara*	Western Coastal Plains and ghats (WCP)	Goa	North Goa	Pro-Indica

138	NKSWR308	*Oryza nivara*	Western Coastal Plains and ghats (WCP)	Goa	North Goa	Pro-Aus
139	NKSWR309	*Oryza spontanea*	Western Coastal Plains and ghats (WCP)	Goa	North Goa	Pro-Aus
140	NKSWR31	*Oryza nivara*	Mid-Gangetic Plains (MGP)	Uttar Pradesh	Chandauli	Pro-Aus
141	NKSWR315	*Oryza nivara*	Western Coastal Plains and ghats (WCP)	Goa	North Goa	Pro-Indica
142	NKSWR316	*Oryza nivara*	Western Coastal Plains and ghats (WCP)	Goa	South Goa	Pro-Aus
143	NKSWR318	*Oryza nivara*	Western Coastal Plains and ghats (WCP)	Goa	South Goa	Pro-Indica
144	NKSWR32	*Oryza nivara*	Mid-Gangetic Plains (MGP)	Uttar Pradesh	Chandauli	Pro-Aus
145	NKSWR329	*Oryza nivara*	Western Coastal Plains and ghats (WCP)	Goa	South Goa	Pro-Aus
146	NKSWR34	*Oryza nivara*	Mid-Gangetic Plains (MGP)	Uttar Pradesh	Chandauli	Pro-Indica
147	NKSWR35	*Oryza nivara*	Mid-Gangetic Plains (MGP)	Uttar Pradesh	Ghazipu	Pro-Aus
148	NKSWR36	*Oryza nivara*	Mid-Gangetic Plains (MGP)	Uttar Pradesh	Ghazipu	Pro-Aus
149	NKSWR362	*Oryza nivara*	Eastern Plateau Hills (EPH)	Chhattisgarh	Kawardha	Pro-Aus
150	NKSWR369	*Oryza nivara*	Eastern Plateau Hills (EPH)	Chhattisgarh	Bilaspur	Mid-Gangatic
151	NKSWR37	*Oryza nivara*	Mid-Gangetic Plains (MGP)	Uttar Pradesh	Ghazipu	Pro-Aus
152	NKSWR38	*Oryza nivara*	Mid-Gangetic Plains (MGP)	Uttar Pradesh	Ghazipu	Pro-Indica
153	NKSWR381	*Oryza rufipogon*	Eastern Himalayan Region (EHR)	Assam	Sibsagar	Pro-Aus
154	NKSWR382	*Oryza nivara*	Eastern Himalayan Region (EHR)	Assam	Golaghat	Pro-Indica
155	NKSWR383	*Oryza rufipogon*	Eastern Himalayan Region (EHR)	Assam	Golaghat	Pro-Aus
156	NKSWR384	*Oryza rufipogon*	Eastern Himalayan Region (EHR)	Assam	Golaghat	Pro-Aus
157	NKSWR386	*Oryza rufipogon*	Eastern Himalayan Region (EHR)	Assam	Golaghat	Pro-Indica
158	NKSWR389	*Oryza rufipogon*	Eastern Himalayan Region (EHR)	Assam	Golaghat	Pro-Indica
159	NKSWR39	*Oryza nivara*	Mid-Gangetic Plains (MGP)	Uttar Pradesh	Ghazipu	Mid-Gangatic
160	NKSWR393	*Oryza rufipogon*	Eastern Himalayan Region (EHR)	Assam	Golaghat	Pro-Indica
161	NKSWR395	*Oryza nivara*	Eastern Himalayan Region (EHR)	Assam	Nagaon	Pro-Indica
162	NKSWR396	*Oryza rufipogon*	Eastern Himalayan Region (EHR)	Assam	Nagaon	Pro-Aus
163	NKSWR397	*Oryza rufipogon*	Western Himalayan Regions (WHR)	Himachal Pradesh	Mandi	Pro-Indica
164	NKSWR399	not assigned	Western Himalayan Regions (WHR)	Himachal Pradesh	Mandi	Pro-Indica
165	NKSWR4	*Oryza rufipogon*	Mid-Gangetic Plains (MGP)	Uttar Pradesh	Mirzapur	Pro-Aus

166	NKSWR401	not assigned	Western Himalayan Regions (WHR)	Himachal Pradesh	Mandi	Pro-Indica
167	NKSWR402	not assigned	Western Himalayan Regions (WHR)	Himachal Pradesh	Mandi	Pro-Indica
168	NKSWR42	*Oryza spontanea*	Mid-Gangetic Plains (MGP)	Bihar	Buxar	Mid-Gangatic
169	NKSWR420	not assigned	Western Himalayan Regions (WHR)	Himachal Pradesh	Kangra	Pro-Indica
170	NKSWR422	not assigned	Western Himalayan Regions (WHR)	Himachal Pradesh	Kangra	Pro-Indica
171	NKSWR423	not assigned	Gujrat Plain and Hills (GPH)	Gujarat	Mehsana	Pro-Indica
172	NKSWR425	not assigned	Gujrat Plain and Hills (GPH)	Gujarat	Mehsana	Pro-Indica
173	NKSWR429	not assigned	Gujrat Plain and Hills (GPH)	Gujarat	Ahmadabad	Pro-Indica
174	NKSWR43	*Oryza nivara*	Mid-Gangetic Plains (MGP)	Bihar	Buxar	Pro-Aus
175	NKSWR430	not assigned	Gujrat Plain and Hills (GPH)	Gujarat	Ahmadabad	Pro-Indica
176	NKSWR432	not assigned	Gujrat Plain and Hills (GPH)	Gujarat	Ahmadabad	Pro-Indica
177	NKSWR436	not assigned	Gujrat Plain and Hills (GPH)	Gujarat	Ahmadabad	Pro-Indica
178	NKSWR438	not assigned	Gujrat Plain and Hills (GPH)	Gujarat	Gandhinagar	Pro-Indica
179	NKSWR439	not assigned	Gujrat Plain and Hills (GPH)	Gujarat	Gandhinagar	Pro-Indica
180	NKSWR44	*Oryza rufipogon*	Mid-Gangetic Plains (MGP)	Uttar Pradesh	Ballia	Mid-Gangatic
181	NKSWR441	not assigned	Mid-Gangetic Plains (MGP)	Uttar Pradesh	Gorakhpur	Pro-Indica
182	NKSWR45	*Oryza nivara*	Mid-Gangetic Plains (MGP)	Uttar Pradesh	Ballia	Pro-Aus
183	NKSWR451	not assigned	Island Regency (IR)	Andaman and nicobar islands	South Andaman	Pro-Indica
184	NKSWR452	not assigned	Island Regency (IR)	Andaman and nicobar islands	Middle Andaman	Pro-Indica
185	NKSWR454	not assigned	Island Regency (IR)	Andaman and nicobar islands	Middle Andaman	Pro-Indica
186	NKSWR455	not assigned	Island Regency (IR)	Andaman and nicobar islands	Middle Andaman	Pro-Indica
187	NKSWR456	not assigned	Island Regency (IR)	Andaman and nicobar islands	North and middle andaman	Pro-Indica
188	NKSWR458	not assigned	Island Regency (IR)	Andaman and nicobar islands	North and middle andaman	Pro-Indica

189	NKSWR46	*Oryza nivara*	Mid-Gangetic Plains (MGP)	Uttar Pradesh	Ballia	Mid-Gangatic
190	NKSWR462	not assigned	Mid-Gangetic Plains (MGP)	Uttar Pradesh	Ballia	Pro-Indica
191	NKSWR464	not assigned	Mid-Gangetic Plains (MGP)	Uttar Pradesh	Ballia	Pro-Indica
192	NKSWR466	not assigned	Mid-Gangetic Plains (MGP)	Uttar Pradesh	Basti	Pro-Indica
193	NKSWR467	not assigned	Mid-Gangetic Plains (MGP)	Uttar Pradesh	Basti	Pro-Indica
194	NKSWR468	not assigned	Mid-Gangetic Plains (MGP)	Uttar Pradesh	MaharajGanj	Pro-Indica
195	NKSWR47	*Oryza nivara*	Mid-Gangetic Plains (MGP)	Uttar Pradesh	Ballia	Mid-Gangatic
196	NKSWR470	not assigned	Mid-Gangetic Plains (MGP)	Uttar Pradesh	MaharajGanj	Pro-Indica
197	NKSWR478	not assigned	Mid-Gangetic Plains (MGP)	Uttar Pradesh	Siddharthnagar	Pro-Indica
198	NKSWR479	not assigned	Mid-Gangetic Plains (MGP)	Uttar Pradesh	Siddharthnagar	Pro-Indica
199	NKSWR47	*Oryza nivara*	Mid-Gangetic Plains (MGP)	Uttar Pradesh	Ballia	Pro-Indica
200	NKSWR483	not assigned	Mid-Gangetic Plains (MGP)	Uttar Pradesh	Siddharthnagar	Pro-Indica
201	NKSWR484	not assigned	Mid-Gangetic Plains (MGP)	Uttar Pradesh	Siddharthnagar	Pro-Indica
202	NKSWR485	not assigned	Mid-Gangetic Plains (MGP)	Uttar Pradesh	Siddharthnagar	Pro-Indica
203	NKSWR486	not assigned	Mid-Gangetic Plains (MGP)	Uttar Pradesh	Balrampur	Pro-Indica
204	NKSWR49	*Oryza nivara*	Mid-Gangetic Plains (MGP)	Uttar Pradesh	Ballia	Mid-Gangatic
205	NKSWR5	*Oryza nivara*	Mid-Gangetic Plains (MGP)	Uttar Pradesh	Mirzapur	Mid-Gangatic
206	NKSWR51	*Oryza spontanea*	Mid-Gangetic Plains (MGP)	Uttar Pradesh	Ballia	Mid-Gangatic
207	NKSWR52	*Oryza nivara*	Mid-Gangetic Plains (MGP)	Uttar Pradesh	Ballia	Mid-Gangatic
208	NKSWR53	*Oryza rufipogon*	Mid-Gangetic Plains (MGP)	Uttar Pradesh	Ballia	Pro-Indica
209	NKSWR54	*Oryza nivara*	Mid-Gangetic Plains (MGP)	Uttar Pradesh	Ballia	Mid-Gangatic
210	NKSWR55	*Oryza spontanea*	Mid-Gangetic Plains (MGP)	Uttar Pradesh	Ballia	Mid-Gangatic
211	NKSWR56	*Oryza nivara*	Mid-Gangetic Plains (MGP)	Uttar Pradesh	Ballia	Mid-Gangatic
212	NKSWR58	*Oryza nivara*	Mid-Gangetic Plains (MGP)	Uttar Pradesh	Ballia	Mid-Gangatic
213	NKSWR59	*Oryza nivara*	Mid-Gangetic Plains (MGP)	Uttar Pradesh	Ballia	Mid-Gangatic
214	NKSWR6	*Oryza nivara*	Mid-Gangetic Plains (MGP)	Uttar Pradesh	Mirzapur	Mid-Gangatic
215	NKSWR60	*Oryza nivara*	Mid-Gangetic Plains (MGP)	Uttar Pradesh	Ballia	Mid-Gangatic
216	NKSWR61	*Oryza nivara*	Mid-Gangetic Plains (MGP)	Uttar Pradesh	RAMPUR	Mid-Gangatic
217	NKSWR62	*Oryza nivara*	Mid-Gangetic Plains (MGP)	Uttar Pradesh	Ballia	Mid-Gangatic

218	NKSWR63	*Oryza nivara*	Mid-Gangetic Plains (MGP)	Uttar Pradesh	Ballia	Mid-Gangatic
219	NKSWR64	*Oryza nivara*	Mid-Gangetic Plains (MGP)	Uttar Pradesh	Mau	Mid-Gangatic
220	NKSWR65	*Oryza spontanea*	Mid-Gangetic Plains (MGP)	Uttar Pradesh	Mau	Mid-Gangatic
221	NKSWR66	*Oryza nivara*	Mid-Gangetic Plains (MGP)	Uttar Pradesh	Mau	Mid-Gangatic
222	NKSWR67	*Oryza nivara*	Mid-Gangetic Plains (MGP)	Uttar Pradesh	Mau	Pro-Aus
223	NKSWR69	*Oryza nivara*	Mid-Gangetic Plains (MGP)	Uttar Pradesh	Mau	Mid-Gangatic
224	NKSWR7	*Oryza nivara*	Mid-Gangetic Plains (MGP)	Uttar Pradesh	Mirzapur	Mid-Gangatic
225	NKSWR71	*Oryza nivara*	Mid-Gangetic Plains (MGP)	Uttar Pradesh	Mau	Mid-Gangatic
226	NKSWR72	*Oryza nivara*	Mid-Gangetic Plains (MGP)	Uttar Pradesh	Azamgarh	Mid-Gangatic
227	NKSWR73	*Oryza nivara*	Mid-Gangetic Plains (MGP)	Uttar Pradesh	Azamgarh	Pro-Aus
228	NKSWR75	*Oryza rufipogon*	Mid-Gangetic Plains (MGP)	Uttar Pradesh	Azamgarh	Mid-Gangatic
229	NKSWR77	*Oryza rufipogon*	Mid-Gangetic Plains (MGP)	Uttar Pradesh	Azamgarh	Mid-Gangatic
230	NKSWR78	*Oryza spontanea*	Mid-Gangetic Plains (MGP)	Uttar Pradesh	Azamgarh	Mid-Gangatic
231	NKSWR79	*Oryza nivara*	Mid-Gangetic Plains (MGP)	Uttar Pradesh	Azamgarh	Pro-Aus
232	NKSWR8	*Oryza nivara*	Mid-Gangetic Plains (MGP)	Uttar Pradesh	Mirzapur	Pro-Aus
233	NKSWR80	*Oryza rufipogon*	Mid-Gangetic Plains (MGP)	Uttar Pradesh	Azamgarh	Pro-Indica
234	NKSWR81	*Oryza nivara*	Mid-Gangetic Plains (MGP)	Uttar Pradesh	Azamgarh	Pro-Indica
235	NKSWR82	*Oryza rufipogon*	Mid-Gangetic Plains (MGP)	Uttar Pradesh	Azamgarh	Mid-Gangatic
236	NKSWR83	*Oryza nivara*	Mid-Gangetic Plains (MGP)	Uttar Pradesh	Azamgarh	Mid-Gangatic
237	NKSWR84	*Oryza spontanea*	Mid-Gangetic Plains (MGP)	Uttar Pradesh	Azamgarh	Mid-Gangatic
238	NKSWR85	*Oryza nivara*	Mid-Gangetic Plains (MGP)	Uttar Pradesh	Azamgarh	Mid-Gangatic
239	NKSWR86	*Oryza rufipogon*	Mid-Gangetic Plains (MGP)	Uttar Pradesh	Azamgarh	Mid-Gangatic
240	NKSWR87	*Oryza nivara*	Mid-Gangetic Plains (MGP)	Uttar Pradesh	Azamgarh	Mid-Gangatic
241	NKSWR88	*Oryza spontanea*	Mid-Gangetic Plains (MGP)	Uttar Pradesh	Mau	Pro-Aus
242	NKSWR89	*Oryza nivara*	Mid-Gangetic Plains (MGP)	Uttar Pradesh	Ghajipur	Mid-Gangatic
243	NKSWR9	*Oryza nivara*	Mid-Gangetic Plains (MGP)	Uttar Pradesh	Mirzapur	Pro-Aus
244	NKSWR90	*Oryza nivara*	Mid-Gangetic Plains (MGP)	Uttar Pradesh	Ghajipur	Pro-Aus
245	NKSWR91	*Oryza spontanea*	Mid-Gangetic Plains (MGP)	Uttar Pradesh	Ghajipur	Pro-Aus
246	NKSWR92	*Oryza nivara*	Mid-Gangetic Plains (MGP)	Uttar Pradesh	Ghajipur	Mid-Gangatic

247	NKSWR93	*Oryza nivara*	Mid-Gangetic Plains (MGP)	Uttar Pradesh	Ghajipur	Mid-Gangatic
248	NKSWR94	*Oryza nivara*	Mid-Gangetic Plains (MGP)	Uttar Pradesh	Jaunpur	Mid-Gangatic
249	NKSWR95	*Oryza nivara*	Mid-Gangetic Plains (MGP)	Uttar Pradesh	Varanashi	Mid-Gangatic
250	NKSWR97	*Oryza nivara*	Mid-Gangetic Plains (MGP)	Uttar Pradesh	Jaunpur	Mid-Gangatic
251	NKSWR98	*Oryza nivara*	Mid-Gangetic Plains (MGP)	Uttar Pradesh	Jaunpur	Pro-Aus
252	NKSWR99	*Oryza nivara*	Mid-Gangetic Plains (MGP)	Uttar Pradesh	Varanashi	Pro-Indica
253	IC330621	*Oryza nivara*	Lower Gangetic Plains (LGP)	West Bengal	Birbhum	Pro-Indica
254	IC330628	*Oryza nivara*	Lower Gangetic Plains (LGP)	West Bengal	24 Parganas	Pro-Indica
255	IC330641	*Oryza nivara*	Lower Gangetic Plains (LGP)	West Bengal	24 Parganas	Pro-Aus
256	IC330643	*Oryza nivara*	Lower Gangetic Plains (LGP)	West Bengal	24 Parganas	Pro-Aus
257	IC330644	*Oryza nivara*	Lower Gangetic Plains (LGP)	West Bengal	24 Parganas	Pro-Aus
258	IC330645	*Oryza nivara*	Lower Gangetic Plains (LGP)	West Bengal	24 Parganas	Pro-Indica
259	IC330646	*Oryza nivara*	Lower Gangetic Plains (LGP)	West Bengal	24 Parganas	Pro-Indica
260	IC330647	*Oryza nivara*	Lower Gangetic Plains (LGP)	West Bengal	24 Parganas	Pro-Indica
261	IC330648	*Oryza nivara*	Lower Gangetic Plains (LGP)	West Bengal	24 Parganas	Pro-Aus
262	IC330649	*Oryza nivara*	Lower Gangetic Plains (LGP)	West Bengal	24 Parganas	Mid-Gangatic
263	IC330650	*Oryza nivara*	Lower Gangetic Plains (LGP)	West Bengal	24 Parganas	Pro-Aus
264	IC330654	*Oryza nivara*	Lower Gangetic Plains (LGP)	West Bengal	Dakshin dinajpur	Pro-Aus
265	IC336687	*Oryza rufipogon*	Eastern Plateau Hills (EPH)	Odisha	Balangir	Pro-Aus
266	IC336712	*Oryza rufipogon*	Eastern Coastal Plains (ECP)	Odisha	Ganjam	Pro-Aus
267	IC336714	*Oryza rufipogon*	Eastern Coastal Plains (ECP)	Odisha	Ganjam	Mid-Gangatic
268	IC336723	*Oryza rufipogon*	Eastern Coastal Plains (ECP)	Odisha	Ganjam	Pro-Indica
269	IC336727	*Oryza rufipogon*	Eastern Plateau Hills (EPH)	Odisha	Khurda	Pro-Indica
270	IC336728	*Oryza rufipogon*	Eastern Plateau Hills (EPH)	Odisha	Khurda	Mid-Gangatic
271	Taipei 309	*Oryza sativa*	Japonica - Improved cultivar			Japonica
272	Pusa 1121	*Oryza sativa*	Indica - Improved cultivar			Aromatic
