# Supplementary material for: A genome-wide association study in Indian wild rice accessions for resistance to the root-knot nematode Meloidogyne graminicola
Source: PLoS One. 2020 Sep 22;15(9):e0239085. doi: 10.1371/journal.pone.0239085 (PMC7508375; doi:10.1371/journal.pone.0239085)
Supplement: S2 Table — Primer Ta− 60°C. RAP-DB: Rice Annotation Project Database (http://rapdb.dna.affrc.go.jp/). RGAP: Rice Genome Annotation Project (http://rice.plantbiology.msu.edu/). (PDF) [file pone.0239085.s007.pdf]

Supplementary Table S2| List of oligonucleotides used for qRT-PCR analysis. Primer T_a – 60°C. RAP-DB: Rice Annotation Project Database (http://rapdb.dna.affrc.go.jp/). RGAP: Rice Genome Annotation Project (http://rice.plantbiology.msu.edu/).

**Gene**	**Locus ID**	**Forward primer (5′-3′)**	**Reverse primer (5′-3′)**	**Putative function**
Actin	RAP-DB: Os03g0718100	CTCTCAGCACATTCCAGCAG	AGGAGGACGGCGATAACAG	Housekeeping gene
QTL 1.1	RGAP: LOC_Os01g11200	TGCGAATTTCCGTAGCTTCT	TCATCCGATACCTCGCAAGT	MYB transcription factor
QTL 1.2	RGAP: LOC_Os01g18440	TGGAGCAGTGCAAGAAGATG	ACGGAGAGGGACTCGTAGGT	MADS-box protein
QTL 2.1	RGAP: LOC_Os02g22140	ATCTCCAGCACGGATTCT	CGTTTCGCTCTGCGTATT	ADP-ribosylation factor
QTL 2.3	RGAP: LOC_Os02g40450	GTTGCCAAATGCATGAGAGA	TTCAGTGCCTTTGCTGTGAC	DEAD/DEAH-box helicase
QTL 3.1	RGAP: LOC_Os03g48450	GCAAGGCAGAGGTTTTGAAG	TCCTTTTGGCTGAGCAACTT	SCARECROW transcription factor
QTL 4.2	RGAP: LOC_Os04g50070	TGTACCCCATCCTGAACTCC	GCAGCTCCAGGTCAATCTTC	C2H2 zinc finger
QTL 4.3	RGAP: LOC_Os04g50660	ATATCCGGGAATGGAACACA	TACAACAGACATCCGGGTCA	WD repeat protein
QTL 6.2	RGAP: LOC_Os06g47260	CTCAAGATCAATGGGGAGGA	GGCAATCATCAAGCCTCCTA	GTP binding Rac protein
QTL 10.1	RGAP: LOC_Os10g33940	GGTTGCTGATCCCAACAGAT	CCTCAAATGGGAAGTCAGGA	Auxin response factor 18
QTL 11.1	RGAP: LOC_Os11g05640	CGCAGTCGTACCAAGAACAG	TTCAGCTTGGTCAGCTCCTT	bZIP transcription factor
QTL 11.2	RGAP: LOC_Os11g10720	CTCAGTGCTCTCGTCCTTCC	TTGCATGTTACCAAGGTCCA	Cf2/Cf5 resistance protein
QTL 11.2	RGAP: LOC_Os11g10760	GCATAAGTGACTGCCCCAAT	TGGTGAAGCAGACTCCACTG	NBS-LRR protein
QTL 11.3	RGAP: LOC_Os11g34450	AGGACATTGCTCTCGCAGAT	TTGGCATCTGAAGTCCACAG	14-3-3 protein
QTL 11.3	RGAP: LOC_Os11g34460	GACGTCACTGTGAGGCTTGA	GCTGCATGTTGACTCCTTCA	F-box protein
