# Supplementary material for: A genome-wide association study in Indian wild rice accessions for resistance to the root-knot nematode Meloidogyne graminicola
Source: PLoS One. 2020 Sep 22;15(9):e0239085. doi: 10.1371/journal.pone.0239085 (PMC7508375; doi:10.1371/journal.pone.0239085)
Supplement: S3 Table — Absolute values represented are the mean of 12 replicates. Forty wild rice genotypes (highlighted in bold letters) that exhibited the lowest number of galls and MF were selected for further studies. Reference genotypes (in bold letters) used was Pusa 1121 and Taipei 309. Most susceptible accessions are highlighted in red color. (PDF) [file pone.0239085.s008.pdf]

Supplementary Table S3| Initial screening of 272 rice accessions for *M. graminicola* resistance in Petri plates containing PF-127 medium at 16 dpi. Absolute values represented are the mean of 12 replicates. Forty wild rice genotypes (highlighted in bold letters) that exhibited the lowest number of galls and MF were selected for further studies. Reference genotypes (in bold letters) used was Pusa 1121 and Taipei 309. Most susceptible accessions are highlighted in red color.

S. No.	Accessions	Species	Agro-climatic zone	Number of galls	Number of endoparasites	Number of egg masses	Number of eggs/egg mass	MF value
1	NKSWR 10	*O. nivara*	MGP	3.88	8.25	5.13	143.90	24.50
**2**	**NKSWR 101**	***O. spontanea***	**MGP**	**0.50**	**0.63**	**0.38**	**2.13**	**0.03**
3	NKSWR102	*O. nivara*	MGP	2.8	4.6	3	115.335	12
4	NKSWR104	*O. nivara*	MGP	0.8	2.9	1.625	62.12375	3.3
5	NKSWR105	*O. nivara*	MGP	3.8	4.3	2.0	72.0	5.0
**6**	**NKSWR 108**	***O. nivara***	**MGP**	**0.38**	**0.88**	**0.38**	**4.00**	**0.05**
7	NKSWR 109	*O. nivara*	MGP	3.63	9.13	6.38	74.95	16.00
**8**	**NKSWR 11**	***O. nivara***	**MGP**	**0.88**	**0.88**	**0.38**	**13.25**	**0.17**
9	NKSWR 110	*O. nivara*	MGP	4.50	8.38	5.88	89.96	18.00
10	NKSWR112	*O. nivara*	MGP	5.125	9.5	6.375	91.6625	20.0
11	**NKSWR113**	***O. nivara***	MGP	**1.75**	**2.25**	**0.625**	**4.125**	**0.15**
**12**	**NKSWR114**	***O. nivara***	**MGP**	**1**	**1.25**	**0.625**	**2.75**	**0.06**
13	NKSWR 115	*O. nivara*	MGP	4.50	7.50	5.25	143.88	25.00
14	NKSWR 116	*O. rufipogon*	MGP	4.13	7.00	4.38	83.01	12.00
15	NKSWR 117	*O. nivara*	MGP	4.00	8.25	5.88	157.19	31.00
16	NKSWR 118	*O. nivara*	MGP	4.38	8.13	5.25	101.25	18.00
17	NKSWR 119	*O. nivara*	MGP	2.63	2.75	1.25	54.87	2.40
18	NKSWR 12	*O. spontanea*	MGP	5.13	9.25	6.25	154.07	32.30
19	NKSWR 120	*O. spontanea*	MGP	4.50	7.50	5.13	67.94	12.00
20	NKSWR 121	*O. nivara*	MGP	3.88	4.13	2.38	70.26	5.60
21	NKSWR 122	*O. nivara*	MGP	2.75	6.00	3.88	64.24	9.00
**22**	**NKSWR 123**	***O. nivara***	**MGP**	**0.38**	**0.50**	**0.13**	**3.50**	**0.01**
**23**	**NKSWR 124**	***O. nivara***	**MGP**	**0.50**	**1.25**	**0.75**	**11.00**	**0.29**
24	NKSWR 125	*O. nivara*	MGP	5.50	9.00	5.75	146.09	28.00
25	NKSWR 126	*O. nivara*	MGP	4.75	4.75	5.25	83.96	15.00
26	NKSWR 127	*O. nivara*	MGP	5.63	10.25	7.63	157.16	40.00
**27**	**NKSWR 128**	***O. nivara***	**MGP**	**0.63**	**1.13**	**0.75**	**13.50**	**0.36**
**28**	**NKSWR 13**	***O. nivara***	**MGP**	**0.88**	**3.13**	**1.63**	**11.75**	**0.62**
29	NKSWR 132	*O. nivara*	MGP	3.50	8.25	6.13	150.58	31.00
30	NKSWR 133	*O. nivara*	MGP	1.63	1.50	0.63	28.42	0.56
31	NKSWR 134	*O. nivara*	MGP	1.88	3.00	1.50	35.79	2.00
32	NKSWR 136	*O. nivara*	MGP	3.63	7.75	5.25	80.94	14.30
33	NKSWR 139	*O. rufipogon*	MGP	3.13	5.88	3.50	75.73	9.00
34	NKSWR 14	*O. nivara*	MGP	3.25	4.25	2.13	34.50	2.40
35	NKSWR 140	*O. nivara*	MGP	4.88	8.25	5.13	89.95	15.00
**36**	**NKSWR 141**	***O. nivara***	**MGP**	**0.75**	**1.38**	**0.75**	**14.13**	**0.40**
37	NKSWR 142	*O. nivara*	MGP	6.25	11.63	7.88	176.32	47.00
**38**	**NKSWR 144**	***O. nivara***	**MGP**	**0.38**	**0.75**	**0.50**	**12.25**	**0.20**
39	NKSWR 145	*O. nivara*	MGP	6.38	11.00	7.63	182.34	46.00
**40**	**NKSWR 146**	***O. rufipogon***	**MGP**	**1.00**	**1.50**	**1.13**	**19.25**	**0.70**
41	NKSWR 148	*O. nivara*	MGP	4.75	11.00	7.63	169.88	43.00
42	NKSWR 149	*O. nivara*	MGP	4.75	11.50	7.88	149.88	40.00

**43**	**NKSWR 15**	***O. nivara***	**MGP**	**0.50**	**1.00**	**0.38**	**8.13**	**0.10**
44	NKSWR 150	*O. nivara*	MGP	4.38	9.00	6.50	88.99	19.00
45	NKSWR 151	*O. rufipogon*	MGP	4.13	10.00	6.50	89.15	19.00
46	NKSWR 152	*O. nivara*	MGP	1.57	2.50	1.38	38.55	1.80
47	NKSWR 153	*O. nivara*	MGP	3.00	3.00	1.50	45.46	2.30
48	NKSWR 154	*O. nivara*	MGP	3.00	4.88	2.88	78.84	8.00
49	NKSWR 155	*O. nivara*	MGP	3.13	3.50	1.88	38.58	2.40
**50**	**NKSWR 156**	***O. nivara***	**MGP**	**0.75**	**1.50**	**0.88**	**13.00**	**0.40**
51	NKSWR 157	*O. nivara*	MGP	2.50	2.75	1.63	36.64	2.00
52	NKSWR 159	*O. nivara*	MGP	3.75	7.88	5.25	88.96	16.00
**53**	**NKSWR 16**	***O. nivara***	**MGP**	**8.00**	**10.25**	**8.88**	**152.60**	**45.20**
**54**	**NKSWR 160**	***O. nivara***	**MGP**	**0.88**	**1.38**	**0.88**	**13.25**	**0.40**
55	NKSWR 161	*O. nivara*	UGP	4.50	9.25	6.38	161.21	34.00
56	NKSWR 162	*O. nivara*	UGP	6.63	11.63	8.13	174.37	47.00
57	NKSWR 163	*O. nivara*	UGP	5.00	11.50	8.13	150.84	41.00
58	NKSWR 164	*O. spontanea*	UGP	4.13	9.50	6.50	102.50	22.00
59	NKSWR 169	*O. nivara*	WHR	4.13	8.38	5.63	74.12	14.00
60	NKSWR 017	*O. spontanea*	MGP	5.13	8.88	5.63	161.32	30.10
61	NKSWR 173	*O. nivara*	WHR	6.88	13.38	9.13	187.65	57.00
62	NKSWR 174	*O. spontanea*	WHR	4.63	8.75	5.88	83.81	17.00
63	NKSWR 179	*O. nivara*	WHR	4.13	7.50	5.38	105.36	19.00
**64**	**NKSWR 18**	***O. nivara***	**MGP**	**0.38**	**0.50**	**0.00**	**0.00**	**0.00**
65	NKSWR 183	*O. nivara*	WHR	4.25	8.50	5.00	83.90	14.00
66	NKSWR 184	*O. spontanea*	WHR	6.00	10.00	7.50	159.49	40.00
67	NKSWR 186	*O. nivara*	WHR	4.00	8.38	5.63	86.53	16.00
68	NKSWR 187	*O. nivara*	UGP	4.75	8.63	6.00	79.56	16.00
69	NKSWR 188	*O. nivara*	UGP	4.63	8.50	5.38	79.30	14.00
70	NKSWR 189	*O. nivara*	UGP	4.50	10.25	6.88	78.71	18.00
**71**	**NKSWR 19**	***O. nivara***	**MGP**	**0.12**	**0.25**	**0.00**	**0.00**	**0.00**
72	NKSWR 190	*O. nivara*	UGP	4.63	9.50	6.63	74.14	16.00
73	NKSWR 191	*O. nivara*	UGP	3.63	8.13	5.38	76.03	14.00
74	NKSWR 192	*O. nivara*	UGP	4.63	8.13	4.88	110.54	18.00
75	NKSWR 196	*O. nivara*	UGP	4.75	8.25	5.25	81.95	14.00
76	NKSWR 197	*O. rufipogon*	UGP	1.75	3.75	2.50	58.39	5.00
77	NKSWR 199	*O. nivara*	UGP	4.25	9.25	6.50	154.54	34.00
78	NKSWR 200	*O. nivara*	UGP	4.625	8.875	5.125	88.7125	15.0
79	NKSWR 202	*O. nivara*	MGP	4.63	9.75	6.63	89.24	20.00
80	NKSWR 205	*O. nivara*	MGP	4.50	8.00	5.75	92.11	18.00
81	NKSWR 207	*O. nivara*	MGP	4.88	8.38	5.50	84.93	16.00
82	NKSWR 21	*O. spontanea*	MGP	3.38	6.63	4.50	74.28	11.14
83	NKSWR 210	*O. nivara*	MGP	4.25	7.75	4.50	88.60	16.00
84	NKSWR218	*O. nivara*	MGP	3.3	4.9	2.5	62.0	5.0
**85**	**NKSWR 22**	***O. nivara***	**MGP**	**1.13**	**3.13**	**1.00**	**3.32**	**0.11**
86	NKSWR 220	*O. nivara*	MGP	4.50	9.00	6.75	142.48	32.00
87	NKSWR 221	*O. nivara*	MGP	3.00	6.63	3.63	116.95	14.00
88	NKSWR 222	*O. nivara*	MGP	2.75	3.75	2.50	50.84	4.20
89	NKSWR 223	*O. nivara*	MGP	4.75	6.88	3.75	142.09	18.00
90	NKSWR 224	*O. nivara*	MGP	3.50	5.50	3.13	104.98	11.00
91	NKSWR 226	*O. nivara*	MGP	5.63	10.00	7.13	156.09	37.00
92	NKSWR 227	*O. nivara*	WCP	3.50	8.75	5.75	145.50	28.00
93	NKSWR 228	*O. nivara*	WCP	4.75	8.25	5.13	102.83	17.00
94	NKSWR 229	*O. nivara*	WCP	3.75	5.25	3.13	75.26	8.00
**95**	**NKSWR 23**	***O. nivara***	**MGP**	**0.63**	**0.75**	**0.25**	**2.25**	**0.02**

96	NKSWR 230	*O. nivara*	WCP	4.13	7.50	5.25	86.19	14.00
97	NKSWR 232	*O. spontanea*	WCP	4.63	10.13	6.88	97.24	22.00
98	NKSWR 233	*O. nivara*	WCP	4.88	9.88	6.63	148.65	32.00
99	NKSWR 234	*O. rufipogon*	WCP	4.38	9.38	6.25	154.90	33.00
100	NKSWR 235	*O. nivara*	WCP	4.38	8.75	5.75	147.56	28.00
101	NKSWR 236	*O. nivara*	WCP	3.50	7.00	4.50	126.38	19.00
102	NKSWR 239	*O. rufipogon*	WCP	2.63	4.50	3.13	78.13	8.00
**103**	**NKSWR 24**	***O. nivara***	**MGP**	**1.00**	**2.25**	**0.63**	**5.75**	**0.11**
104	NKSWR 241	*O. nivara*	WCP	3.25	5.25	3.38	88.57	10.00
105	NKSWR 242	*O. nivara*	WCP	6.75	11.75	7.63	178.15	45.00
106	NKSWR 243	*O. nivara*	WHR	5.00	10.38	7.63	170.59	43.00
107	NKSWR 245	*O. nivara*	WHR	5.13	10.13	7.13	150.48	35.00
108	NKSWR 246	*O. spontanea*	WHR	4.13	8.38	6.00	123.10	25.00
109	NKSWR 247	*O. spontanea*	WHR	4.00	8.38	5.25	126.79	22.00
**110**	**NKSWR 248**	***O. spontanea***	**WHR**	**13.00**	**31.63**	**22.25**	**82.03**	**61.00**
111	NKSWR 249	*O. nivara*	WHR	5.25	9.50	6.50	132.15	29.00
**112**	**NKSWR 25**	***O. nivara***	**MGP**	**0.38**	**0.75**	**0.25**	**2.50**	**0.25**
113	NKSWR 250	*O. spontanea*	WHR	2.75	6.00	3.00	105.00	11.00
114	NKSWR 251	*O. nivara*	GPH	4.75	7.63	5.13	117.38	20.00
115	NKSWR 252	*O. nivara*	GPH	7.63	10.63	7.00	171.89	42.00
116	NKSWR 253	*O. spontanea*	GPH	2.38	4.25	2.50	82.08	7.00
117	NKSWR 254	*O. nivara*	GPH	4.00	7.13	4.38	111.68	17.00
118	NKSWR 255	*O. nivara*	GPH	4.63	7.25	4.50	109.24	16.00
119	NKSWR 257	*O. nivara*	GPH	6.00	9.75	6.25	143.11	30.00
120	NKSWR 258	*O. nivara*	GPH	4.50	6.75	4.38	63.44	9.00
**121**	**NKSWR 259**	***O. nivara***	**GPH**	**0.12**	**0.12**	**0.00**	**0.00**	**0.00**
122	NKSWR 260	*O. nivara*	GPH	5.50	9.63	6.50	137.43	30.00
123	NKSWR 261	*O. spontanea*	GPH	4.50	7.63	5.38	110.88	20.00
124	NKSWR 262	*O. nivara*	GPH	2.63	3.25	1.50	35.58	1.80
125	NKSWR 263	*O. spontanea*	GPH	5.13	6.88	4.25	130.05	19.00
126	NKSWR 264	*O. nivara*	GPH	3.63	5.50	3.75	107.60	14.00
127	NKSWR 265	*O. nivara*	GPH	3.38	6.63	4.00	86.60	11.00
128	NKSWR 269	*O. spontanea*	GPH	4.00	5.75	3.50	83.45	10.00
129	NKSWR 27	*O. spontanea*	MGP	7.38	10.38	6.50	158.22	34.20
130	NKSWR 28	*O. spontanea*	MGP	3.50	9.25	5.63	159.11	29.60
**131**	**NKSWR 29**	***O. nivara***	**MGP**	**1.25**	**2.75**	**1.38**	**4.13**	**0.19**
132	NKSWR 3	*O. nivara*	MGP	5.38	10.25	6.63	145.68	32.00
**133**	**NKSWR 30**	***O. nivara***	**MGP**	**0.00**	**0.00**	**0.00**	**0.00**	**0.00**
134	NKSWR 302	*O. nivara*	WCP	5.38	8.50	6.00	89.20	18.00
135	NKSWR 304	*O. nivara*	WCP	4.63	7.00	4.75	81.54	13.00
136	NKSWR 306	*O. spontanea*	WCP	5.50	9.50	5.88	121.86	24.00
137	NKSWR 307	*O. nivara*	WCP	6.00	10.63	6.75	120.83	27.00
138	NKSWR 308	*O. nivara*	WCP	4.75	7.63	4.75	85.94	14.00
139	NKSWR 309	*O. spontanea*	WCP	4.88	7.25	4.50	81.93	12.00
**140**	**NKSWR 31**	***O. nivara***	**MGP**	**1.13**	**2.00**	**1.13**	**7.38**	**0.27**
141	NKSWR 315	*O. nivara*	WCP	2.88	3.13	1.75	56.78	3.50
142	NKSWR 316	*O. nivara*	WCP	4.75	7.50	4.63	84.38	13.00
143	NKSWR 318	*O. nivara*	WCP	5.38	10.25	6.50	99.64	21.00
144	NKSWR 32	*O. nivara*	MGP	4.38	8.88	5.50	147.03	0.27
145	NKSWR 329	*O. nivara*	WCP	5.63	9.13	6.13	130.96	27.00
146	NKSWR 34	*O. nivara*	MGP	1.00	3.88	2.13	36.38	2.50
**147**	**NKSWR 35**	***O. nivara***	**MGP**	**1.38**	**2.75**	**0.88**	**6.33**	**0.18**
**148**	**NKSWR 36**	***O. nivara***	**MGP**	**1.25**	**3.50**	**1.63**	**6.13**	**0.32**

149	NKSWR 362	*O. nivara*	EPH	3.88	8.88	5.00	84.81	14.00
150	NKSWR 369	*O. nivara*	EPH	3.38	5.75	3.25	89.98	10.00
**151**	**NKSWR 37**	***O. nivara***	**MGP**	**1.63**	**2.38**	**1.63**	**17.63**	**0.93**
**152**	**NKSWR 38**	***O. nivara***	**MGP**	**1.38**	**2.63**	**1.75**	**15.38**	**0.92**
153	NKSWR 381	*O. rufipogon*	EHR	4.63	7.63	4.88	81.65	13.00
154	NKSWR 382	*O. nivara*	EHR	4.75	7.00	4.75	83.13	13.00
155	NKSWR 383	*O. rufipogon*	EHR	4.88	8.63	5.88	85.50	17.00
156	NKSWR 384	*O. rufipogon*	EHR	4.75	8.13	5.50	78.69	15.00
**157**	**NKSWR 386**	***O. rufipogon***	**EHR**	**5.00**	**8.63**	**6.38**	**82.75**	**18.00**
158	NKSWR 389	*O. rufipogon*	EHR	4.63	8.75	5.75	68.19	13.00
159	**NKSWR 39**	***O. nivara***	**MGP**	**1.75**	**2.75**	**2.13**	**27.25**	**1.91**
160	NKSWR 393	*O. rufipogon*	EHR	4.63	8.13	5.63	75.50	14.00
161	NKSWR 395	*O. nivara*	EHR	5.00	8.00	5.63	95.38	18.00
162	NKSWR 396	*O. rufipogon*	EHR	4.75	7.25	4.38	83.79	12.00
163	NKSWR 397	*O. rufipogon*	WHR	2.63	3.00	1.75	32.98	2.00
164	NKSWR 399	not assigned	WHR	4.50	9.13	7.25	118.00	29.00
165	NKSWR 4	*O. rufipogon*	MGP	4.25	6.50	3.88	86.55	11.20
166	NKSWR 401	not assigned	WHR	5.88	9.63	6.88	125.06	29.00
167	NKSWR 402	not assigned	WHR	7.50	10.75	8.00	160.35	43.00
168	NKSWR 42	*O. spontanea*	MGP	5.00	7.63	5.00	148.90	24.80
169	NKSWR 420	not assigned	WHR	2.75	3.50	1.38	36.41	1.70
170	NKSWR 422	not assigned	WHR	2.38	4.75	2.38	43.23	3.40
171	NKSWR 423	not assigned	GPH	2.25	5.13	2.88	49.92	5.00
172	NKSWR 425	not assigned	GPH	2.88	7.00	4.25	77.21	11.00
173	NKSWR 429	not assigned	GPH	3.50	8.25	5.88	79.66	16.00
**174**	**NKSWR 43**	***O. nivara***	**MGP**	**0.38**	**0.38**	**0.38**	**0.38**	**0.00**
175	NKSWR 430	not assigned	GPH	1.50	1.13	0.50	13.28	0.21
176	NKSWR 432	not assigned	GPH	3.25	6.88	4.38	74.56	11.00
177	NKSWR 436	not assigned	GPH	2.25	5.75	2.75	47.08	4.40
178	NKSWR 438	not assigned	GPH	2.38	4.13	2.13	42.13	3.00
179	NKSWR 439	not assigned	GPH	2.38	5.00	2.63	46.88	4.00
**180**	**NKSWR 44**	***O. rufipogon***	**MGP**	**1.50**	**2.25**	**1.38**	**9.63**	**0.58**
181	NKSWR 441	not assigned	MGP	5.75	10.38	6.75	88.01	20.00
**182**	**NKSWR 45**	***O. nivara***	**MGP**	**1.13**	**1.88**	**1.13**	**16.13**	**0.60**
183	NKSWR 451	not assigned	IR	4.75	8.75	6.00	84.34	17.00
184	NKSWR 452	not assigned	IR	4.00	8.50	5.50	79.71	15.00
185	NKSWR 454	not assigned	IR	3.63	7.38	4.88	72.93	12.00
186	NKSWR 455	not assigned	IR	3.63	6.63	3.63	75.41	9.00
187	NKSWR 456	not assigned	IR	5.25	9.00	5.88	93.50	18.00
188	NKSWR 458	not assigned	IR	4.75	8.13	5.13	95.40	15.00
189	NKSWR 46	*O. nivara*	MGP	3.13	4.63	2.25	67.24	5.15
190	NKSWR 462	not assigned	MGP	4.25	8.00	5.25	84.26	15.00
191	NKSWR 464	not assigned	MGP	3.75	6.38	4.50	94.73	14.00
192	NKSWR 466	not assigned	MGP	4.13	7.88	5.25	80.65	14.00
193	NKSWR 467	not assigned	MGP	4.50	7.88	5.38	82.95	14.00
194	NKSWR 468	not assigned	MGP	3.88	7.25	5.13	74.55	13.00
195	NKSWR 47	*O. nivara*	MGP	2.63	4.38	3.13	140.15	14.50
196	NKSWR 470	not assigned	MGP	3.75	7.88	5.38	79.34	14.00
197	NKSWR 478	not assigned	MGP	3.38	6.50	4.00	72.84	10.00
198	NKSWR 479	not assigned	MGP	4.25	7.38	4.38	79.09	12.00
**199**	**NKSWR 48**	***O. nivara***	**MGP**	**0.80**	**0.80**	**0.40**	**12.90**	**0.17**
200	NKSWR 483	not assigned	MGP	5.50	9.25	6.50	76.90	16.00
201	NKSWR 484	not assigned	MGP	5.13	10.00	7.38	89.03	22.00

202	NKSWR 485	not assigned	MGP	5.63	10.13	6.75	78.60	17.00
203	NKSWR 486	not assigned	MGP	4.75	6.63	3.75	75.88	10.00
204	NKSWR 49	*O. nivara*	MGP	7.25	10.00	7.63	180.89	46.00
**205**	**NKSWR 5**	***O. nivara***	**MGP**	**1.75**	**2.13**	**1.50**	**17.94**	**0.89**
**206**	**NKSWR 51**	***O. spontanea***	**MGP**	**1.25**	**1.25**	**1.25**	**17.13**	**0.74**
207	NKSWR 52	*O. nivara*	MGP	5.63	8.00	5.25	141.11	25.00
208	NKSWR 53	*O. rufipogon*	MGP	4.63	9.50	6.38	94.76	20.20
**209**	**NKSWR 54**	***O. nivara***	**MGP**	**1.25**	**1.38**	**0.88**	**16.13**	**0.48**
**210**	**NKSWR 55**	***O. spontanea***	**MGP**	**1.13**	**1.13**	**0.88**	**15.25**	**0.45**
211	NKSWR 56	*O. nivara*	MGP	7.13	13.75	11.63	161.42	62.40
212	NKSWR 58	*O. nivara*	MGP	6.00	9.00	5.88	137.65	27.00
213	NKSWR 59	*O. nivara*	MGP	5.88	7.38	5.00	152.24	25.30
214	NKSWR 6	*O. nivara*	MGP	5.00	8.13	5.00	93.30	15.50
215	NKSWR 60	*O. nivara*	MGP	4.75	7.25	5.00	148.11	24.66
216	NKSWR 61	*O. nivara*	MGP	4.88	8.00	5.63	156.16	29.00
217	NKSWR 62	*O. nivara*	MGP	4.38	6.88	5.13	135.85	23.00
218	NKSWR 63	*O. nivara*	MGP	5.13	7.75	5.63	148.29	27.60
219	NKSWR 64	*O. nivara*	MGP	3.38	4.00	2.00	51.70	3.44
220	NKSWR 65	*O. spontanea*	MGP	3.13	7.50	5.63	107.17	20.00
221	NKSWR 66	*O. nivara*	MGP	5.00	9.75	6.25	101.14	21.23
222	NKSWR 67	*O. nivara*	MGP	3.25	7.25	4.13	111.02	15.10
223	NKSWR 69	*O. nivara*	MGP	6.63	9.00	6.00	154.79	31.00
224	NKSWR 7	*O. nivara*	MGP	5.00	7.88	5.00	138.05	23.00
225	NKSWR 71	*O. nivara*	MGP	4.63	9.13	5.88	77.05	15.16
226	NKSWR 72	*O. nivara*	MGP	2.88	7.75	5.63	93.22	17.40
227	NKSWR 73	*O. nivara*	MGP	3.75	6.50	4.38	109.13	16.00
228	NKSWR 75	*O. rufipogon*	MGP	3.63	5.13	3.50	115.66	13.50
229	NKSWR 77	*O. rufipogon*	MGP	4.00	6.38	4.50	124.37	19.00
230	NKSWR 78	*O. spontanea*	MGP	3.88	6.63	4.88	134.49	22.00
231	NKSWR 79	*O. nivara*	MGP	4.00	8.13	6.13	140.71	29.00
232	NKSWR 8	*O. nivara*	MGP	4.75	9.50	6.88	83.99	19.30
233	NKSWR 80	*O. rufipogon*	MGP	4.75	7.75	5.75	133.54	26.00
234	NKSWR 81	*O. nivara*	MGP	6.25	8.25	5.88	144.06	28.00
235	NKSWR 82	*O. rufipogon*	MGP	3.00	4.75	3.00	130.14	13.00
236	NKSWR 83	*O. nivara*	MGP	5.00	8.00	5.00	79.19	13.00
237	NKSWR 84	*O. spontanea*	MGP	5.38	8.00	4.88	82.11	14.00
238	NKSWR 85	*O. nivara*	MGP	4.00	6.13	4.50	146.98	22.00
239	NKSWR 86	*O. rufipogon*	MGP	3.25	8.63	5.63	89.81	17.00
240	NKSWR 87	*O. nivara*	MGP	5.00	10.00	7.75	190.19	49.40
241	NKSWR 88	*O. spontanea*	MGP	4.63	8.50	5.75	181.37	35.00
242	NKSWR 89	*O. nivara*	MGP	4.25	8.50	5.63	101.40	19.00
**243**	**NKSWR 9**	***O. nivara***	**MGP**	**1.25**	**2.50**	**1.38**	**16.85**	**0.78**
244	NKSWR 90	*O. nivara*	MGP	3.50	7.38	4.88	188.62	31.00
245	NKSWR 91	*O. spontanea*	MGP	2.75	5.50	4.00	158.31	21.00
246	NKSWR 92	*O. nivara*	MGP	1.75	3.00	1.63	84.86	4.50
247	NKSWR 93	*O. nivara*	MGP	5.38	8.50	6.13	166.17	34.00
248	NKSWR 94	*O. nivara*	MGP	2.88	4.50	3.25	85.57	9.40
249	NKSWR 95	*O. nivara*	MGP	4.00	8.25	6.00	133.58	27.00
250	NKSWR 97	*O. nivara*	MGP	3.50	4.88	3.50	106.73	13.00
251	NKSWR 98	*O. nivara*	MGP	5.13	11.13	7.63	152.29	39.00
252	NKSWR 99	*O. nivara*	MGP	4.25	9.25	5.88	72.30	14.00
253	IC330621	*O. nivara*	LGP	1.5	3.8	1.8	32.4	2.0
254	IC330628	*O. nivara*	LGP	3.8	4.8	3.0	66.5	7

255	IC330641	*O. nivara*	LGP	4.63	7.63	4.75	81.51	13.00
256	IC330643	*O. nivara*	LGP	2.88	3.00	1.75	31.38	2.00
257	IC330644	*O. nivara*	LGP	5.25	7.38	4.13	90.49	12.00
258	IC330645	*O. nivara*	LGP	3.25	3.75	2.38	82.84	7.00
259	IC330646	*O. nivara*	LGP	4.50	5.50	3.75	70.85	9.00
260	IC330647	*O. nivara*	LGP	4.8	6.9	4.5	76.1	11
261	IC330648	*O. nivara*	LGP	5.0	8.9	5.8	77.7	15
262	IC330649	*O. nivara*	LGP	2.88	4.63	2.75	79.04	7.00
263	IC330650	*O. nivara*	LGP	5.63	9.25	5.88	87.29	17.00
264	IC330654	*O. nivara*	LGP	3.6	5.4	2.8	71.4	6.6
**265**	**IC336687**	***O. rufipogon***	**EPH**	**0.50**	**1.6**	**0.5**	**11.7**	**0.195**
266	IC336712	*O. rufipogon*	ECP	4.50	8.63	5.63	73.94	14.00
267	IC336714	*O. rufipogon*	ECP	4.63	6.88	4.75	72.63	12.00
268	IC336723	*O. rufipogon*	ECP	4.6	7.8	5.1	81.0	13
269	IC336727	*O. rufipogon*	EPH	3.9	8.4	5.1	81.2	14
270	IC336728	*O. rufipogon*	EPH	3.88	6.75	4.00	65.88	9.00
**271**	**Taipei 309**	***O. sativa***	**-**	**7.00**	**20.50**	**12.50**	**89.25**	**37.10**
**272**	**Pusa 1121**	***O. sativa***	**-**	**7.88**	**16.38**	**14.38**	**102.44**	**49.15**
