# Supplementary material for: A genome-wide association study in Indian wild rice accessions for resistance to the root-knot nematode Meloidogyne graminicola
Source: PLoS One. 2020 Sep 22;15(9):e0239085. doi: 10.1371/journal.pone.0239085 (PMC7508375; doi:10.1371/journal.pone.0239085)
Supplement: S4 Table — Absolute values represented are the mean of 12 replicates ± standard error. The reference genotypes used were Pusa 1121 and Taipei 309. (PDF) [file pone.0239085.s009.pdf]

Supplementary Table S4| Screening of 40 rice accessions for *M. graminicola* resistance in pots containing soil at 16 dpi. Absolute values represented are the mean of 12 replicates ± standard error. The reference genotypes used were Pusa 1121 and Taipei 309.

S. No.	Accessions	Species	Agro-climatic zone	Number of galls	Number of endoparasites	Number of egg masses	Number of eggs/egg mass	MF value
1	NKSWR 101	*O. spontanea*	MGP	4.3 ± 0.65	5.2 ± 0.75	5.5 ± 0.75	52.5 ± 5.5	1.44 ± 0.18
2	NKSWR 108	*O. nivara*	MGP	1.5 ± 0.25	2.5 ± 0.45	2.6 ± 0.36	25.5 ± 3.60	0.33 ± 0.09
3	NKSWR 11	*O. nivara*	MGP	6.5 ± 1.2	8.9 ± 2.2	9.2 ± 1.8	75.6 ± 6.9	3.48 ± 0.55
4	NKSWR113	*O. nivara*	MGP	10.3 ± 1.5	14.6 ± 2.45	12.4 ± 2.8	88.5 ± 7.5	5.49 ± 0.95
5	NKSWR114	*O. nivara*	MGP	7.6 ± 1.38	8.5 ± 1.65	11.3 ± 2.5	82.1± 7.25	4.64 ± 0.85
6	NKSWR 123	*O. nivara*	MGP	1.3 ± 0.18	2.2 ± 0.25	2.5 ± 0.12	26 ± 3.5	0.33 ± 0.08
7	NKSWR 124	*O. nivara*	MGP	3.9 ± 0.5	4.8 ± 0.65	5.2 ± 1.18	49.4 ± 5.66	1.28 ± 0.22
8	NKSWR 128	*O. nivara*	MGP	5.3 ± 0.6	6.2 ± 1.15	5.5 ± 0.65	55.6 ± 5.62	1.53 ± 0.25
9	NKSWR 13	*O. nivara*	MGP	10.5 ± 1.2	13.4 ± 2.9	12.9 ± 2.7	94.5 ± 3.69	6.1± 0.95
10	NKSWR 141	*O. nivara*	MGP	9.8 ± 1.18	11.3 ± 1.5	10.9 ± 2.2	43.5 ± 5.68	2.37 ± 0.48
11	NKSWR 144	*O. nivara*	MGP	3.5 ± 0.65	3.9 ± 0.40	3.5 ± 0.6	29.6 ± 5.88	0.52 ± 0.1
12	NKSWR 146	*O. rufipogon*	MGP	8.8 ± 1.2	10.5 ± 1.5	8.9 ± 1.45	85.7 ± 5.34	3.81 ± 0.75
13	NKSWR 15	*O. nivara*	MGP	5.5 ± 0.95	5.8 ± 1.44	5.5 ± 1.26	73.5 ± 5.92	2.02 ± 0.4
14	NKSWR 156	*O. nivara*	MGP	4.5 ± 0.45	5.2 ± 0.60	4.8 ± 0.37	76.3 ± 5.78	1.83 ± 0.2
15	NKSWR 160	*O. nivara*	MGP	5.4 ± 0.65	6.5 ± 0.65	6 ± 1.09	84.5 ± 5.35	2.54 ± 0.55
16	NKSWR 18	*O. nivara*	MGP	1.9 ± 0.26	2.3 ± 0.32	1.9 ± 0.2	30.4 ± 3.5	0.29 ± 0.09
17	NKSWR 19	*O. nivara*	MGP	1.4 ± 0.11	1.5 ± 0.22	1.2 ± 0.1	33.6 ± 4.5	0.2 ± 0.07
18	NKSWR 22	*O. nivara*	MGP	8.5 ± 0.85	9.5 ± 1.47	8.6 ± 1.64	98.6 ± 9.74	4.24 ± 0.85
19	NKSWR 23	*O. nivara*	MGP	6.6 ± 0.76	7.2 ± 1.3	6.5 ± 1.8	102.5 ± 11.8	3.33 ± 0.75
20	NKSWR 24	*O. nivara*	MGP	8.2 ± 0.88	9.4 ± 1.77	8.3 ± 1.26	99.5 ± 11.5	4.13 ± 0.85
21	NKSWR 25	*O. nivara*	MGP	1.7 ± 0.28	1.9 ± 0.41	1.9 ± 0.28	33.2 ± 3.7	0.32 ± 0.09
22	NKSWR 259	*O. nivara*	GPH	0.25 ± 0.1	0.12 ± 0.12	0.5 ± 0.1	19.75 ± 2.5	0.05 ± 0.03
23	NKSWR 29	*O. nivara*	MGP	9.3 ± 1.33	9.5 ± 1.81	9.5 ± 2.5	95.5 ± 10.4	4.54 ± 1.15
24	NKSWR 30	*O. nivara*	MGP	0.25 ± 0.05	0.25 ± 0.05	0.25 ± 0.1	15.25 ± 1.15	0.02 ± 0.01
25	NKSWR 31	*O. nivara*	MGP	8.7 ± 1.25	9 ± 1.95	9 ± 1.64	100.2 ± 10.8	4.51 ± 1.15
26	NKSWR 35	*O. nivara*	MGP	10.5 ± 1.5	11.3 ± 1.95	10.6 ± 2.25	105.7 ± 11.5	5.6 ± 1.45
27	NKSWR 36	*O. nivara*	MGP	9.1 ± 1.15	10 ± 1.25	9.5 ± 1.55	110.5 ± 11.8	5.25 ± 1.35
28	NKSWR 37	*O. nivara*	MGP	10.2 ± 1.55	10.5 ± 1.65	10.8 ± 1.65	111.6 ± 12.5	6.03 ± 1.75
29	NKSWR 38	*O. nivara*	MGP	9.8 ± 1.45	11. 3± 2.06	10.2 ± 2.25	106.7 ± 9.9	5.44 ± 1.55
30	NKSWR 39	*O. nivara*	MGP	12.5 ± 1.75	15.5 ± 1.65	13.6 ± 2.5	122.5 ± 13.5	8.33 ± 2.25
31	NKSWR 43	*O. nivara*	MGP	0.9 ± 0.2	2.2 ± 0.38	1.5 ± 0.25	29.8 ± 4.8	0.22 ± 0.06
32	NKSWR 44	*O. rufipogon*	MGP	10 ± 0.95	11.4 ± 1.85	10.2 ± 2.45	115 ± 12.2	5.87 ± 1.75
33	NKSWR 45	*O. nivara*	MGP	9.7 ± 0.95	10.8 ± 1.65	9.9 ± 2.15	112.5 ± 12.4	5.57 ± 1.65
34	NKSWR 48	*O. nivara*	MGP	7.6 ± 1.25	7.9 ± 1.35	7.9 ± 1.18	93.8 ± 10.88	3.71 ± 1.1
35	NKSWR 5	*O. nivara*	MGP	10.6 ± 1.4	14.9 ± 2.5	11.1 ± 1.5	109.7 ± 10.9	6.09 ± 1.85
36	NKSWR 51	*O. spontanea*	MGP	9.2 ± 1.15	10.2 ± 1.45	9.3 ± 1.4	99.6 ± 9.9	4.63 ± 1.5
37	NKSWR 54	*O. nivara*	MGP	8.8 ± 0.95	8.5 ± 1.05	8.8 ± 1.15	104.8 ± 10.2	4.61 ± 1.5
38	NKSWR 55	*O. spontanea*	MGP	8.4 ± 0.9	8.8 ± 1.15	8.3 ± 1.28	105.5 ± 10.72	4.38 ± 1.35
39	NKSWR 9	*O. nivara*	MGP	9.2 ± 1.73	9.5 ± 1.92	9.2 ± 1.53	112.4 ± 9.9	5.17 ± 1.6

40	IC 336687	*O. rufipogon*	EPH	1.8 ± 0.18	3.5 ± 0.86	2.5 ± 0.75	39.6 ± 6.40	0.5 ± 0.1
41	Taipei 309	*O. sativa*	-	39.6 ± 2.38	44.5 ± 2.55	45.7 ± 2.62	254.6 ± 15.66	58.18 ± 5.5
42	Pusa 1121	*O. sativa*	-	41.5 ± 3.44	43.4 ± 2.77	47.8 ± 2.76	255.8 ± 17.78	61.14 ± 5.95
