# Supplementary material for: A genome-wide association study in Indian wild rice accessions for resistance to the root-knot nematode Meloidogyne graminicola
Source: PLoS One. 2020 Sep 22;15(9):e0239085. doi: 10.1371/journal.pone.0239085 (PMC7508375; doi:10.1371/journal.pone.0239085)
Supplement: Attachment — Submitted filename: Revision.docx [file pone.0239085.s010.docx]

General comments: The paper is interesting and provides novel valuable information regarding new sources of genetic resistance in rice. My main concern regards the RT-qPCR experiments. It is not clear at all how the authors calculated the relative expression values of the selected genes. In papers reporting rigorous RT-qPCR experiments is not accepted to use only one reference gene, but at least three reference genes have to be included. I can understand that is often hard to find three or more stable reference gene, thus, in the case the authors decide to use only one reference gene, they have to demonstrate that this gene is stable under the experimental conditions. In this case the authors have to show the reaction efficiency and R^2^ and Cq values of the reference genes under the different experimental conditions. Moreover, the relative expression values are calculated by normalizing the Cq value of one gene to the Cq value of the reference gene and the Cq values of the mock control (and the relative expression values of the gene is calculated as follows: relative exp value= 2^-ΔΔCq^). In this manuscript it seems that the authors normalized the Cq values of the selected gene only to the Cq value of the reference genes, thus it is not clear at all how they calculated the relative expression value. I suggest to carefully read this paper: Bustin et al. 2009 “The MIQE guidelines: minimum information for publication of quantitative real-time PCR experiments”, Clinical Chemistry 55:4 611-622.

Please also the check the quality of your figures, some graphs are not clear, difficult to read.

Please change qRT-PCR into RT-*q*PCR

Line 219 “RNA quantity and quality were assessed in Nanodrop ND-1000 spectrophotometer”. To check the RNA quality is essential to run an agarose gel to verify if the extracted RNA was degraded or not. The quantification by using the Nanodrop does not provide information about the integrity of the RNA. Please, provide the gel of RNA as supplementary materials

Line 220 Please specify the starting amount of RNA used to synthetized the cDNA
