# Supplementary material for: A genome-wide association study in Indian wild rice accessions for resistance to the root-knot nematode Meloidogyne graminicola
Source: PLoS One. 2020 Sep 22;15(9):e0239085. doi: 10.1371/journal.pone.0239085 (PMC7508375; doi:10.1371/journal.pone.0239085)
Supplement: Attachment — Submitted filename: Rebuttal letter.docx [file pone.0239085.s011.docx]

**Rebuttal letter**

**Reviewer #1:**

General comments: My main concern regards the RT-qPCR experiments. It is not clear at all how the authors calculated the relative expression values of the selected genes. In papers reporting rigorous RT-qPCR experiments is not accepted to use only one reference gene, but at least three reference genes have to be included. I can understand that is often hard to find three or more stable reference gene, thus, in the case the authors decide to use only one reference gene, they have to demonstrate that this gene is stable under the experimental conditions. In this case the authors have to show the reaction efficiency and R2 and Cq values of the reference genes under the different experimental conditions. Moreover, the relative expression values are calculated by normalizing the Cq value of one gene to the Cq value of the reference gene and the Cq values of the mock control (and the relative expression values of the gene is calculated as follows: relative exp value= 2-ΔΔCq). In this manuscript it seems that the authors normalized the Cq values of the selected gene only to the Cq value of the reference genes, thus it is not clear at all how they calculated the relative expression value. I suggest to carefully read this paper: Bustin et al. 2009 “The MIQE guidelines: minimum information for publication of quantitative real-time PCR experiments”, Clinical Chemistry 55:4 611-622.
Please also the check the quality of your figures, some graphs are not clear, difficult to read.
Please change qRT-PCR into RT-qPCR
Line 219 “RNA quantity and quality were assessed in Nanodrop ND-1000 spectrophotometer”. To check the RNA quality is essential to run an agarose gel to verify if the extracted RNA was degraded or not. The quantification by using the Nanodrop does not provide information about the integrity of the RNA. Please, provide the gel of RNA as supplementary materials
Line 220 Please specify the starting amount of RNA used to synthetized the cDNA.

**Response:**

We have revised qPCR figure (now Figure 8 in revised version) using *O. sativa* actin as the reference gene. The relative quantities of each candidate gene transcript in the samples were calculated via 2^-ΔCq^ [ΔCq = (Cq for candidate gene) – (Cq for actin)] method (Schmittgen and Livak, 2008; Nature Protocols 3:1101-1108). Obtained value in mock inoculated at 0 days were transformed into 1 and with identical conversion factor expression value of mock and nematode-inoculated at 7 days was transformed and statistically compared. Similar qPCR analysis method was used by Warmerdam et al. 2018, New Phytologist 218:724-737; Warmerdam et al. 2019, Molecular Plant Pathology 20:137-152.

As warranted by the esteemed reviewer we have now added a supplementary table depicting the expression stability of reference gene (actin) in different experimental conditions such as leaf, root, stem and seed tissues of resistant and susceptible genotype in mock and nematode inoculated condition. qRT-PCR is changed to RT-qPCR in the text of revised MS.

Line 219. Gel of RNA is given in supplementary figure S.

Line 220. Starting amount of RNA was 500 ng.

**Reviewer #3:**

It should be described the relationships between 40 resistant accessions and genotypes of significant SNPs detected. Therefore, I recommend the authors to add genotype data of the accessions and to discuss about the contribution of detected QTLs to RRKN resistant accessions. Additionally, I found some points that needed to revise or confirm. I recommend the authors to check these points carefully.
1) Relationship between resistant accessions and genotypes of significant SNPs should be described.
2) p.8, l. 226. “Table S4” should be changed to “Table S2”.
3) p.11, l. 311-312. According to the Fig. 3, the r values in the text seem to represent r square values. It should be changed to the values of correlation coefficients.
4) p.12, l. 337-338. I am confusing because you mentioned that population I and III are designated as “Pro-Aus” and “Pro-Indica”, respectively. And population II was designated as “Mid-Gangetic”. However, in the subsequent sentences, percentages of admixture types do not match with Fig. 4b. I wonder that population I, II and III are designated as “Pro-Aus”, “Pro-Indica” and “Mid-Gangetic”, respectively.
5) p.14, l. 394. “Table S2” should be changed to “Table S1”.
6) p.17, l. 431. “Os11g34450” should be changed to “Os11g34460”.
7) p.18, l. 443. “12 candidate genes” should be changed to “14 candidate genes”.
8) p.20, l. 523-525. I don’t understand the meaning of the sentence. I recommend you to rewrite it.
9) p.22, l. 557. “Rac protein; [70]; Moeder et al. [71]” should be changed to “Rac protein; [70, 71]”.

**Response:**

1) We have described the Relationship between resistant accessions and genotypes of significant SNPs in supplementary figure 5.

2) p.8, l. 226. Suggested change is effected in the revised MS.

3) p.11, l. 311-312. Suggested change is effected in the revised MS.

4) p.12, l. 337-338. Indeed, population I, II and III should be designated as “Pro-Aus”, “Pro-Indica” and “Mid-Gangetic”, respectively. We have rectified the mistake in revised MS.

5) p.14, l. 394. Suggested change is effected in the revised MS.

6) p.17, l. 431. Suggested change is effected in the revised MS.

7) p.18, l. 443. Suggested change is effected in the revised MS.

8) p.20, l. 523-525. The sentence conveys that since RRKN is not a pest of japonica rice, it has not undergone any selection process to inherit nematode resistance. Thus, japonica rice might have shown nematode susceptibility in our study.

9) p.22, l. 557. Suggested change is effected in the revised MS.
